# Inflammation causes remodeling of mitochondrial cytochrome *c* oxidase mediated by the bifunctional gene *C15orf48*

**DOI:** 10.1126/sciadv.abl5182

**Published:** 2021-12-08

**Authors:** Sally A. Clayton, Kalbinder K. Daley, Lucy MacDonald, Erika Fernandez-Vizarra, Giovanni Bottegoni, John D. O’Neil, Triin Major, Daniel Griffin, Qinqin Zhuang, Adeolu B. Adewoye, Kieran Woolcock, Simon W. Jones, Carl Goodyear, Aziza Elmesmari, Andrew Filer, Daniel A. Tennant, Stefano Alivernini, Christopher D. Buckley, Robert D. S. Pitceathly, Mariola Kurowska-Stolarska, Andrew R. Clark

**Affiliations:** 1 Research into Inflammatory Arthritis Centre Versus Arthritis (RACE), Universities of Glasgow, Birmingham, Newcastle, Oxford, UK.; 2 Institute of Inflammation and Ageing, University of Birmingham, Birmingham, UK.; 3 Institute of Metabolism and Systems Research, University of Birmingham, Birmingham, UK.; 4 Institute of Infection, Immunity and Inflammation, University of Glasgow, Glasgow, UK.; 5 Institute of Molecular, Cell and Systems Biology, University of Glasgow, Glasgow, UK.; 6 Dipartimento di Scienze Biomolecolari, University of Urbino, Urbino, Italy.; 7 School of Pharmacy, Institute of Clinical Sciences, University of Birmingham, Birmingham, UK.; 8 Division of Rheumatology, Fondazione Policlinico Universitario A. Gemelli IRCCS, Rome, Italy.; 9 Kennedy Institute of Rheumatology, University of Oxford, Oxford, UK.; 10 Department of Neuromuscular Diseases, UCL Queen Square Institute of Neurology and The National Hospital for Neurology and Neurosurgery, London, UK.

## Abstract

Dysregulated mitochondrial function is a hallmark of immune-mediated inflammatory diseases. Cytochrome *c* oxidase (C*c*O), which mediates the rate-limiting step in mitochondrial respiration, is remodeled during development and in response to changes of oxygen availability, but there has been little study of C*c*O remodeling during inflammation. Here, we describe an elegant molecular switch mediated by the bifunctional transcript *C15orf48*, which orchestrates the substitution of the C*c*O subunit NDUFA4 by its paralog C15ORF48 in primary macrophages. Expression of *C15orf48* is a conserved response to inflammatory signals and occurs in many immune-related pathologies. In rheumatoid arthritis, *C15orf48* mRNA is elevated in peripheral monocytes and proinflammatory synovial tissue macrophages, and its expression positively correlates with disease severity and declines in remission. *C15orf48* is also expressed by pathogenic macrophages in severe coronavirus disease 2019 (COVID-19). Study of a rare metabolic disease syndrome provides evidence that loss of the NDUFA4 subunit supports proinflammatory macrophage functions.

## Teaser

Exchange of mitochondrial subunits C15ORF48 and NDUFA4 occurs during macrophage activation in vitro and in inflammatory disease.

## INTRODUCTION

Changes in activation state of immune cells are accompanied by, and dependent on, profound alterations of cellular metabolism (*1*, *2*). Mitochondria are versatile organelles that play central roles in energy metabolism and act as signaling hubs. The ability of mitochondria to shape cellular phenotype has become a key concept in the field of immunometabolism (*3*). An example of mitochondrial plasticity is the reprogramming of myeloid cells in response to activation or polarization cues. Stimulation of murine macrophages and dendritic cells with the pathogen-associated molecular pattern lipopolysaccharide (LPS) results in a Warburg-like metabolic state involving rapid up-regulation of glycolytic metabolism and lactate production. Mitochondria in these cells are described as being repurposed for the generation of reactive oxygen species (ROS), which have both signaling and direct bactericidal roles, as well as for the diversion of tricarboxylic acid (TCA) cycle metabolites into biosynthetic and immunoregulatory pathways (*4*–*6*). However, there is growing evidence that metabolic responses to proinflammatory stimuli differ between human and mouse myeloid cells, suggesting that conclusions based on mouse cells should be extrapolated with caution (*7*). It has been demonstrated that metabolic pathways and mitochondrial activity contribute to autoimmune and chronic inflammatory pathologies such as rheumatoid arthritis (RA), underpinning the concept that these conditions might be effectively treated by targeting cellular metabolism (*8*–*10*).

Mitochondrial adaptation occurs on several different scales (*11*–*14*), and disruption of these processes is a common hallmark of many inflammatory, neurodegenerative, oncological, and age-related diseases (*15*). Mitochondrial regulation at a gross scale includes the balancing of biogenesis and mitophagy to control mitochondrial number and quality, the opposing processes of fusion and fission, and architectural organization of mitochondrial membranes and formation of cristae (*16*, *17*). The complexes of the mitochondrial electron transport chain associate with each other forming supercomplexes, the assembly and turnover of which may represent an additional level at which mitochondrial activity is regulated (*18*, *19*). At a smaller scale, mitochondrial function may be fine-tuned by remodeling of individual electron transport chain complexes (*20*). This is particularly evident in the case of complex IV [the cytochrome *c* oxidase (C*c*O) complex], a multiprotein complex that transfers electrons from cytochrome *c* to molecular oxygen as the final and rate-limiting step of the electron transport chain (*21*). This complex is the only one within the electron transport chain that contains tissue-specific isoforms of several subunits (*20*). The hypoxia-responsive transcription factor hypoxia-inducible factor-1α (HIF-1α) mediates interchange of C*c*O subunits in response to changing availability of oxygen (*20*, *22*–*24*).

NDUFA4 [reduced form of nicotinamide adenine dinucleotide (NADH) dehydrogenase (ubiquinone) 1α subcomplex subunit 4] was originally identified as a subunit of electron transport chain complex I. More recent evidence indicates that NDUFA4 is a component of the C*c*O complex and is essential for maximal C*c*O enzymatic activity in muscle and HeLa cells (*25*–*28*). Very rare homozygous loss-of-function mutations of the *NDUFA4* gene causes a severe neuromuscular condition resembling Leigh syndrome, characterized by impairment of C*c*O activity in muscle, lactic acidosis, dystonia, ataxia, and spasticity (*26*). A recent crystallographic structure of human C*c*O, isolated using the mild detergent digitonin, showed NDUFA4 to be located at the periphery of the complex, as a transmembrane protein, making contacts with the mitochondrial membrane lipid cardiolipin (*28*). NDUFA4 is thought to be added late in the biogenesis of the C*c*O complex, its peripheral site accounting for its facile loss under certain conditions of mitochondrial isolation (*18*, *29*). Renaming it as COXFA4 (cytochrome c oxidase subunit FA4) has been proposed but not yet formally adopted (*27*). A recently identified form of electron transport chain subunit exchange may occur between NDUFA4 and the related protein NDUFA4L2. Increased NDUFA4L2 expression and reciprocal down-regulation of NDUFA4 occur under hypoxia and are associated with poor prognosis in patients with cancer (*30*–*32*). NDUFA4L2 expression has been shown to aid cell survival and proliferation by reducing oxidative stress (*30*, *32*–*34*). However, it has not yet been formally shown whether NDUFA4L2 replaces NDUFA4 in C*c*O.

The *C15orf48* gene is otherwise known as *NMES1* in human and as *Nmes1* or *AA467197* in mouse. Until relatively recently, the main insight into the role of this gene came from the observations that the gene locus is highly methylated in epithelial cell cancers, resulting in markedly reduced transcript expression (*35*, *36*). The gene encodes a short protein product with poorly described function. In addition, the *C15orf48/NMES1* gene encodes the microRNA (miRNA) miR-147b (miR-147 in mouse), which is up-regulated in response to Toll-like receptor (TLR) activation of macrophages and has been reported to indirectly down-regulate expression of inflammatory cytokines interleukin-6 (IL-6) and tumor necrosis factor–α (TNFα) (*37*). More recently, the C15ORF48 protein has been described to be mitochondrially localized and was found to interact with subunits of complexes I, III, and IV (*38*).

Although much is known about metabolic reprogramming in activated myeloid cells (*2*, *5*), the process of mitochondrial electron transport chain component switching is poorly understood in this context. Here, we describe an elegant and economical mechanism by which a single bifunctional transcript causes substitution of the C*c*O component NDUFA4 by the related protein C15ORF48. This substitution is a conserved response to many different inflammatory challenges both in vitro and in vivo. Expression of *C15orf48* is elevated in peripheral monocytes and synovial macrophages of patients with RA. Its expression forms part of a coherent metabolic reprograming, correlates positively with disease severity, and declines in remission. In severe coronavirus disease 2019 (COVID-19), *C15orf48* is highly expressed by pathogenic populations of macrophages that invade the airways. We also show that macrophages from individuals lacking NDUFA4 display a hyperinflammatory phenotype, characterized by elevated chemokine production, linking this regulatory axis to potentially pathogenic mechanisms during inflammatory disease.

## RESULTS

### The highly conserved gene *C15orf48* and its miRNA product are induced during macrophage stimulation

**Fig. 1.**
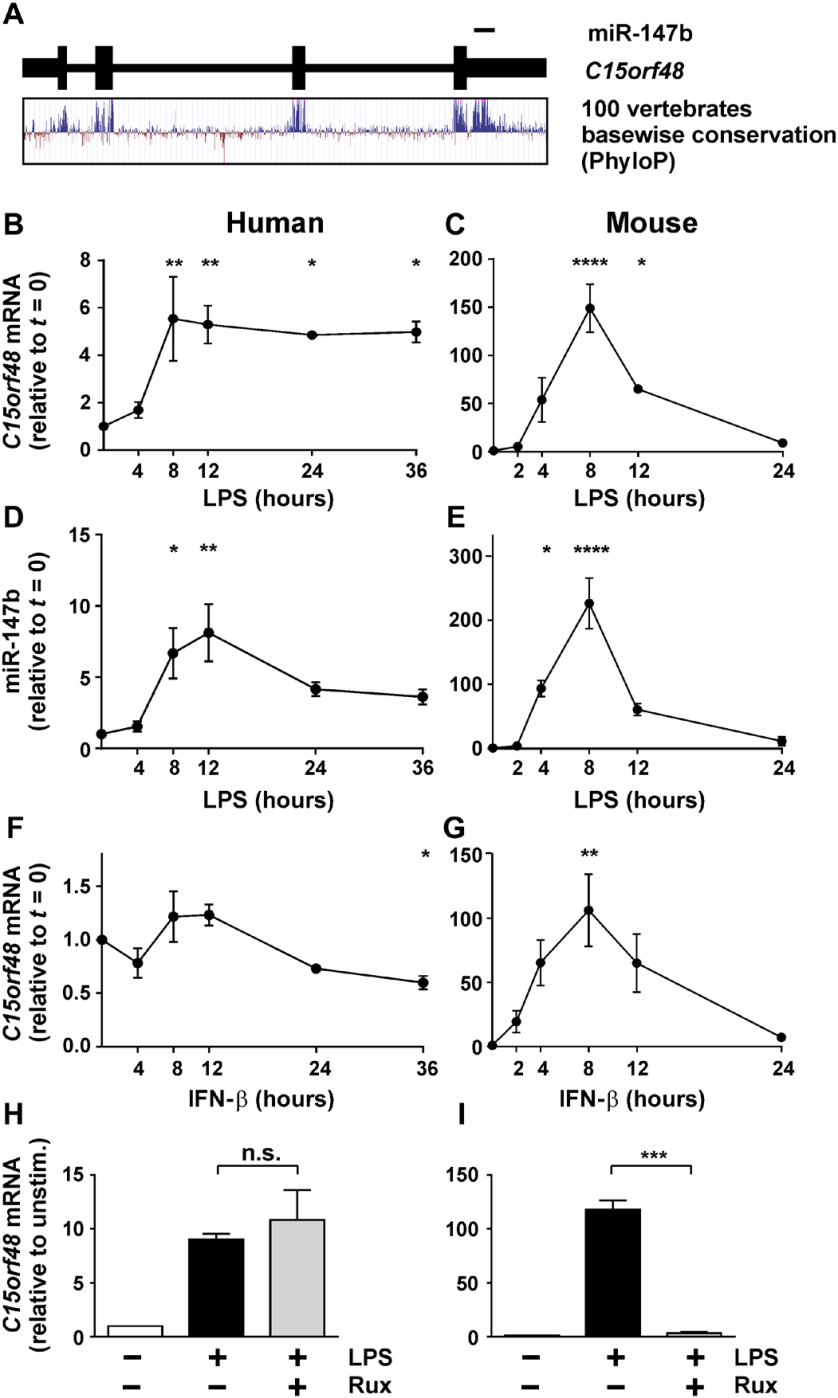
*C15orf48* expression is induced by LPS treatment of human and mouse macrophages. (**A**) Basewise conservation of the *C15orf48* gene, including miR-147b encoding sequence, across vertebrate species based on PhyloP analysis (UCSC Genome Browser, https://genome.ucsc.edu). (**B** to **I**) Expression of *C15orf48* transcript or mature miR-147b in human peripheral blood MDMs and mouse BMDMs detected by reverse transcription quantitative polymerase chain reaction (RT-qPCR) (all *n* = 3; means ± SEM). (B to E) Cells treated with LPS (10 ng/ml) for the indicated times. (F and G) Cells treated with IFN-β (10 ng/ml) for the indicated times. (H and I) Cells treated with LPS (10 ng/ml) with or without Rux (1 μM) for 12 hours (human, left) or 8 hours (mouse, right). (B to G) One-way analysis of variance (ANOVA) with Dunnett’s correction for multiple comparisons relative to time 0. (H and I) Two-tailed *t* test. *P < 0.05; **P < 0.01; ***P < 0.001; ****P < 0.0001. n.s., not significant.

The *C15orf48* transcript, transcribed from chromosome 15 in humans, encodes a small protein of 83 amino acids. It is also the source of miRNA-147b-3p (hereafter referred to as miR-147b), which is produced from the 3′ untranslated region (3′UTR) encoded by the fourth exon. Both the open reading frame and miRNA source sequence are very highly conserved (Fig. 1A). For example, the latter is identical in human, cow, hen, and green anole lizard *C15orf48* transcripts. In the orthologous mRNAs of coelacanth, a lobe-finned fish, and six randomly selected ray-finned fish, we found a maximum of two nucleotide differences within the miR-147b source sequence, none within the seed sequence that is the principal determinant of target specificity. This suggests that the miRNA has been subject to strong selective pressure for more than 450 million years. Expression of mature *C15orf48* mRNA and mature miR-147b was increased by LPS treatment of primary human monocyte-derived macrophages (MDMs) (Fig. 1, B and D). Both the mRNA and its miRNA product were even more strongly induced by LPS in mouse bone marrow–derived macrophages (BMDMs) (Fig. 1, C and E), consistent with a previous report (*37*). According to publicly available microarray datasets, *C15orf48* was among the most highly up-regulated genes in several inflammatory pathologies including sepsis, intracerebral hemorrhage, psoriasis, and RA (Table 1).

**Table 1.** Expression of *C15orf48* mRNA in selected gene expression datasets. Inspected using GEO2R at GEO (http://ncbi.nlm.nih.gov/geo). Log_2_FC, log_2_ fold change; NA, not applicable.

**GSE (GEO series reference)**	**Comparison**	**Log_2_FC**	**Centile**	***P*_adj_**	**Centile**
77298	Synovial biopsy; RA versus healthy control	3.956	100	0.0498	100
7307	Synovial biopsy; RA versus healthy control	3.966	100	0.0084	100
14905	Skin biopsy; lesional psoriatic versus noninvolved	2.8	100	9.91 × 10^–13^	100
24265	Brain biopsy; perihematoma (stroke) versus contralateral	4.604	100	0.135	93
16538	Lung biopsy; pulmonary fibrosis versus healthy control	3.01	100	0.13211	91
28619	Liver biopsy; alcoholic hepatitis versus healthy control	3.56	100	3.49 × 10^–5^	97
64486	Coronary artery biopsy; Kawasaki’s disease versus control	2.0	NA	0.0004	NA
40885	Alveolar macrophages after instillation of LPS versus PBS	3.42	100	0.00032	92
48119	Whole blood 4 hours after LPS injection versus 0 hours	3.383	100	8.86 × 10^–16^	100
48080	PBMCs of patients with sepsis at diagnosis versus healthy controls	2.567	100	0.454	100
46955	Peripheral monocytes of patients with sepsis versus healthy controls	2.5	100	2.01 × 10^–4^	98
28991	Whole blood; early dengue infection versus recovery	4.296	100	3.66 × 10^–9^	100

Analysis of publicly available expression data highlighted polyinosinic:polycytidylic acid [poly(I:C)] and type 1 interferons (IFNs) as strong inducers of *C15orf48* expression in murine macrophages. However, these responses were notably reduced or absent in human MDMs (table S1). We confirmed that IFN-β is a potent inducer of *C15orf48* in mouse BMDMs (Fig. 1G). Janus kinase–signal transducer and activator of transcription (JAK-STAT) signaling was necessary for LPS-induced expression in mouse BMDMs as the response could be blocked by the Janus kinase inhibitor ruxolitinib (Rux) (Fig. 1I), suggesting that secondary signaling through the type 1 IFN receptor controls *C15orf48* expression in these cells. Consistent with this hypothesis, the expression of *C15orf48* in response to lipid A (a purified TLR4 agonist) was strongly impaired by disruption of mouse *Ifnar1*, *Trif*, or *Irf3* genes, all of which play essential roles in IFN-β signaling (*C15orf48* is identified by its alternative name AA467197 in this paper) (*39*). In contrast, human *C15orf48* expression was not induced by IFN-β (Fig. 1F), and its LPS-induced expression was insensitive to Rux (Fig. 1H). These results indicate that while the sequences of this gene and its miRNA are highly conserved, the signaling pathways controlling their expression are divergent.

### miR-147b targets electron transport chain component *NDUFA4*

**Fig. 2.**
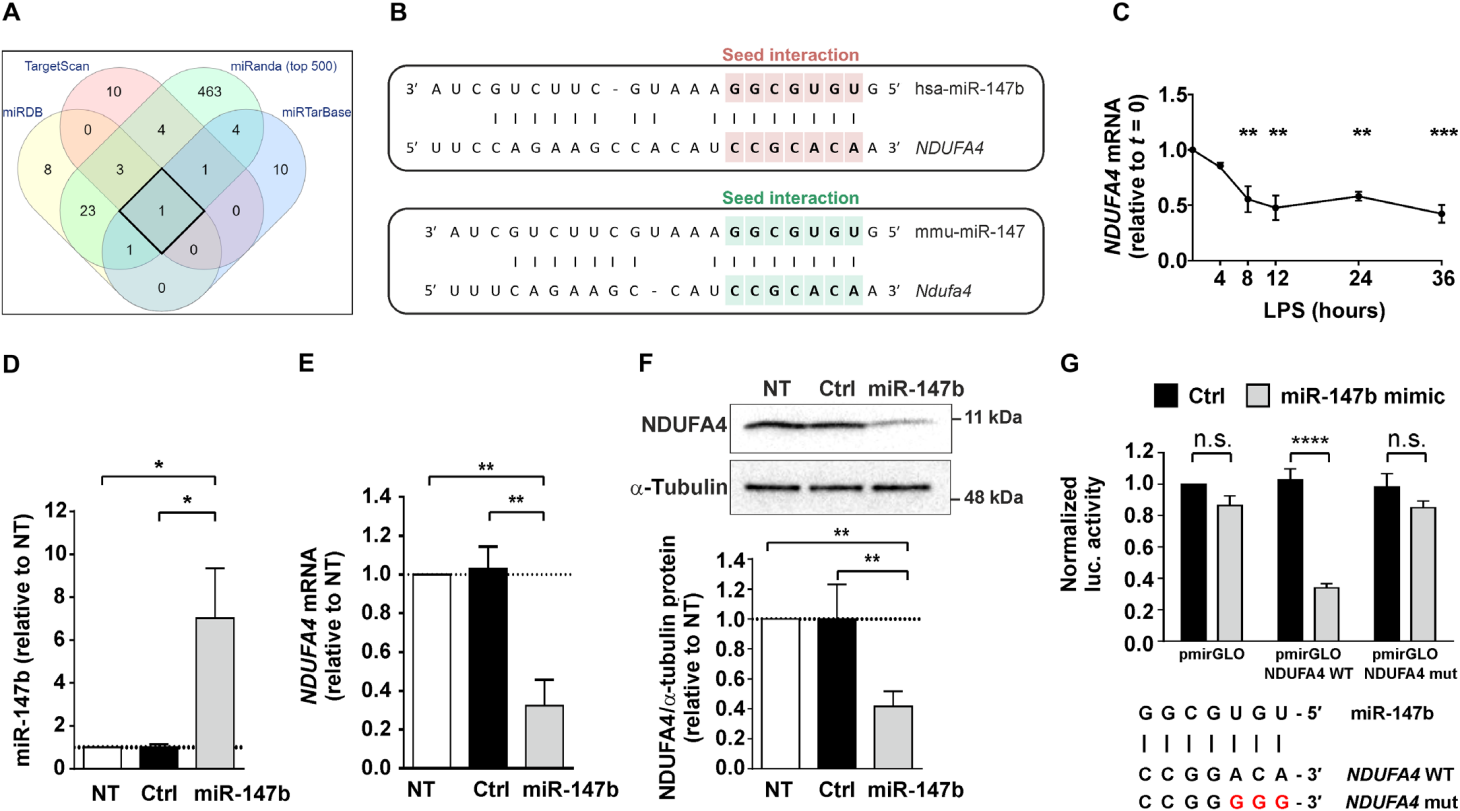
miR-147b targets electron transport chain component gene *NDUFA4*. (**A**) Comparison of human miR-147b target prediction results from four online tools: mirDB, TargetScan, miRTarBase, and miRanda (top 500 results only from miRanda used for comparison). (**B**) Schematic of predicted target match between 3′UTR of *NDUFA4/Ndufa4* and miR-147b/miR-147 in human and mouse. miRNA seed sequence highlighted, showing 100% match to predicted target site. (**C**) *NDUFA4* mRNA expression in human MDMs treated with LPS (10 ng/ml) for the indicated times (*n* = 3; means ± SEM). (**D** to **F**) Human MDMs transfected with control (Ctrl) or miR-147b mimic (2 nM), or nontransfected (NT). miR-147b (D) or *NDUFA4* mRNA (E) detected by RT-qPCR (*n* = 3; means ± SEM). NDUFA4 protein (F) detected by Western blotting. Representative blot and quantification by densitometry normalized to α-tubulin, reported relative to nontransfected condition (*n* = 3; means ± SD). (**G**) miRNA luciferase reporter assay performed in human embryonic kidney (HEK) 293 cells using unmodified reporter plasmid (pmirGLO), reporter plasmid containing wild-type (WT) *NDUFA4* 3′UTR (pmirGLO NDUFA4 WT) and reporter plasmid containing mutated *NDUFA4* 3′UTR (pmirGLO NDUFA4 mut) (*n* = 3; means ± SEM). Schematic shows miRNA seed sequence and corresponding target sequence in WT and mutated plasmids. (C) One-way ANOVA with Dunnett’s correction for multiple comparisons relative to time 0. (D to F) One-way ANOVA with Tukey’s correction for multiple comparisons. (G) Two-way ANOVA with Sidak correction for multiple comparisons. *P < 0.05; **P < 0.01; ***P < 0.001; ****P < 0.0001.

Four online algorithms were used to predict mRNA targets of miR-147b: TargetScan7.1, miRTarBase, miRDB, and miRanda. *NDUFA4* was the only target predicted by all four algorithms (Fig. 2A) and was the top scoring target in each case. Interaction between miR-147b and murine *Ndufa4* mRNA was conserved, although with some differences of base pairing outside of the seed sequence (Fig. 2B), and was also predicted by the algorithms listed above. LPS treatment of MDMs caused sustained down-regulation of *NDUFA4* mRNA (Fig. 2C). A miR-147b mimic was transfected into primary human MDMs, with careful titration to achieve a level similar to that in LPS-treated MDMs to minimize off-target effects (Fig. 2D). The miR-147b mimic significantly reduced the expression of both *NDUFA4* mRNA and NDUFA4 protein (Fig. 2, E and F). A luciferase reporter containing the *NDUFA4* 3′UTR was down-regulated by miR-147b mimic cotransfection but not by a negative control miRNA. Mutation of the putative seed binding sequence rendered the *NDUFA4* reporter nonresponsive to the miR-147b mimic (Fig. 2G). These results confirm a conserved, seed-dependent interaction between miR-147b and *NDUFA4* mRNA.

### C15ORF48 protein replaces NDUFA4 within C*c*O under inflammatory conditions

**Fig. 3.**
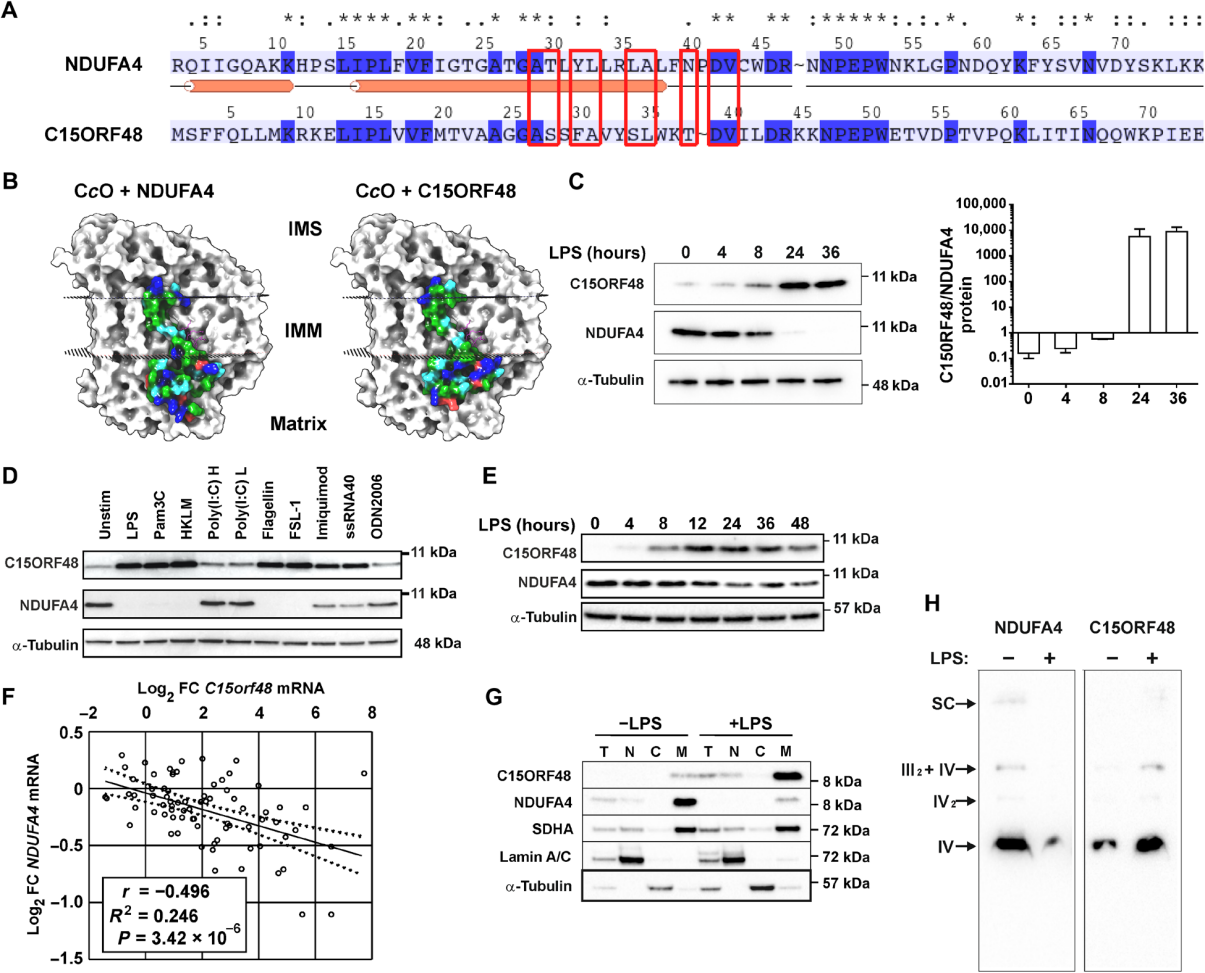
C15ORF48 protein exchanges with NDUFA4 within C*c*O in response to TLR activation. (**A**) Pairwise amino acid alignment between NDUFA4 (positions 3 to 76 of 81) and C15ORF48 (positions 1 to 74 of 83). Identical positions (dark blue and *); conserved residues (:); similar residues (.); residues predicted to directly contact cardiolipin (red boxes). Secondary structure as solved in (*28*) (orange tubes, α helix). (**B**) Comparison of solved structure of NDUFA4 (left) with modeled structure of C15ORF48 (right) complexed with cardiolipin and C*c*O subunits. Connolly surface of NDUFA4 and C15orf48 in complex-forming conformations cultured according to residue type: blue, positively charged; red, negative; green, hydrophobic. Connolly surface of other subunits in light gray. Cardiolipin in ball-and-stick representation (magenta carbon atoms). Mitochondrial membrane highlighted by dotted surfaces. IMM, inner mitochondrial membrane; IMS, inter-membrane space. (**C**) C15ORF48 and NDUFA4 protein detected by Western blotting from human MDMs LPS treated (10 ng/ml) for the indicated times. Quantification by densitometry, expressed as C15ORF48/NDUFA4 ratio (*n* = 3; means ± SD). (**D**) C15ORF48 and NDUFA4 protein detected by Western blotting from human MDMs treated with the indicated TLR agonists for 24 hours. Pam3C, Pam3CSK4; HKLM, heat-killed *Listeria monocytogenes*; Poly(I:C) H, high molecular weight; Poly(I:C) L, low molecular weight; ODN2006, class B CpG oligonucleotide. Representative of *n* = 2. (**E**) C15ORF48 and NDUFA4 protein detected by Western blotting from mouse BMDMs treated with LPS (10 ng/ml) for the indicated times. Representative of *n* = 6. (**F**) Regression analysis of *C15orf48* and *NDUFA4/Ndufa4* differential expression in unstimulated versus stimulated human and mouse macrophages. Combined from 10 datasets [Gene Expression Omnibus (GEO)], in which a range of stimuli were used (see Materials and Methods). FC, fold change. (**G**) Human MDMs unstimulated or LPS treated (10 ng/ml) for 24 hours, separated into nuclear (N), cytosolic (C), or mitochondrial (M) fractions. T, total cell lysate. Blotted for C15ORF48, NDUFA4, mitochondrial, nuclear and cytosolic markers SDHA, lamin A/C, and α-tubulin, respectively. Representative of *n* = 3. (**H**) Blue native polyacrylamide gel electrophoresis (BN-PAGE) and Western blotting to determine NDUFA4 and C15ORF48 distribution in electron transport chain (ETC) complexes and supercomplexes. Human MDMs unstimulated or LPS treated (10 ng/ml) for 24 hours. Representative of *n* = 4.

In the UniProt database, C15ORF48 protein is annotated as a relative of NDUFA4. Amino acid identity between C15ORF48 and NDUFA4 is 26%, but the two proteins display high conservation of amino acid properties (fig. S1, A and B) and matching predicted α-helical transmembrane regions (Fig. 3A). The topology of NDUFA4 within the C*c*O complex is known from a published cryo–electron microscopy (cryo-EM) structure (*28*). In this three-dimensional model, NDUFA4 could be substituted by C15ORF48 without any conflicts (Fig. 3B). Predicted interactions between NDUFA4 and cardiolipin within the inner mitochondrial membrane (IMM) were partially conserved. In the hypothetical structure, the regions of sequence divergence between the two proteins projected outward from the C*c*O complex on the matrix side of the IMM (Fig. 3B). A systematic analysis of protein-protein interactions within mitochondria (*38*) identified four structural components of the C*c*O complex (COX5A, COX6A1, COX7C, and COX8A) that interact with both NDUFA4 and C15ORF48 but none that interact uniquely with either NDUFA4 or C15ORF48. No interactions between NDUFA4 and C15ORF48 were detected, suggesting that occupancy of C*c*O by the two proteins is mutually exclusive.

In human MDMs stimulated with LPS, there was notable inverse regulation of NDUFA4 and C15ORF48 protein levels over a 36-hour time course (Fig. 3C). Several other TLR ligands also increased C15ORF48 and decreased NDUFA4 protein levels (Fig. 3D). Notable exceptions were poly(I:C) and ODN2006, which activate the endosomally localized receptors TLR3 and TLR9, respectively. This result is consistent with the transcriptomic analysis that showed little or no response of *C15orf48* or *NDUFA4* genes to poly(I:C) in human macrophages (table S1). Imiquimod and single-stranded RNA caused relatively weak up-regulation of C15ORF48 protein and partial loss of NDUFA4 protein. In mouse BMDMs, LPS also caused inverse regulation of NDUFA4 and C15ORF48 protein levels, although the loss of NDUFA4 was less marked than observed in human primary macrophages (Fig. 3E). Inspection of publicly accessible macrophage gene expression datasets revealed that *C15orf48* expression was commonly increased in both mouse and human primary macrophages by proinflammatory stimuli such as purified TLR agonists or exposure to pathogens (Fig. 3F). The response of the *C15orf48* gene to these different stimuli was consistently ranked above the 90th centile in terms of both magnitude and statistical significance (table S1). In the same datasets, the expression of *Ndufa4*/*NDUFA4* mRNA was generally decreased, although with smaller fold changes (Fig. 3F). Changes in the expression of *C15orf48* and *Ndufa4*/*NDUFA4* mRNA were significantly negatively correlated across these varied experimental conditions (*P* = 3.42 × 10^−6^), suggesting a mechanistic link and a conserved response to several pathogen-related challenges.

Fractionation of untreated or LPS-stimulated primary human MDMs confirmed that both NDUFA4 and C15ORF48 proteins were predominantly localized to mitochondria (tracks marked “M” in Fig. 3G). One-dimensional blue native polyacrylamide gel electrophoresis (BN-PAGE) was then performed on mitochondrially enriched lysates of control and LPS-treated MDMs. Under resting conditions, Western blotting confirmed the presence of NDUFA4 in all C*c*O-containing structures (based on well-established migration patterns) (Fig. 3H). Specifically, NDUFA4 was present in C*c*O (complex IV) monomers, dimers, III_2_ + IV supercomplexes, and higher-order I + III_2_ + IV supercomplexes. Consistent with the expression data, the overall levels of NDUFA4 protein and amounts within each C*c*O-containing species were decreased following LPS treatment (Fig. 3H). C15ORF48 was detected in all of the same electron transport chain complexes and supercomplexes but showed the reverse pattern of expression, being increased in response to LPS (Fig. 3H). No band corresponding to the size of “free” complex I could be detected with either anti-NDUFA4 or anti-C15ORF48 antibodies under resting or LPS-stimulated conditions.

LPS-activated mouse macrophages enter a pseudo-hypoxic state, in which HIF-1α becomes activated despite the presence of adequate oxygen (*40*, *41*). In some solid tumors, hypoxia promotes down-regulation of NDUFA4 and reciprocal up-regulation of NDUFA4L2, at least partly mediated by HIF-1α (*30*, *32*, *34*, *42*). We therefore asked whether the expression of NDUFA4L2 was influenced by LPS treatment of MDMs. We were unable to detect *NDUFA4L2* mRNA in primary human macrophages activated by LPS, subjected to hypoxia or treated with the hypoxia mimetic dimethyloxallylglycine (fig. S2A), despite the fact that all of these treatments caused robust activation of HIF-1α (fig. S2B). Hypoxia-treated HeLa cells robustly increased *NDUFA4L2* mRNA as previously reported (*34*), providing a positive control for this experiment (fig. S2A). In primary human macrophages, hypoxia partially decreased NDUFA4 protein levels, but this response occurred in the absence of any detectable change in expression of C15ORF48 protein (fig. S2C). Therefore, the substitution of NDUFA4 by C15ORF48 in macrophages is a specific response to proinflammatory stimuli and not a consequence of the pseudo-hypoxic state.

### An additional, miR-147b–independent mechanism of down-regulation of NDUFA4 protein

**Fig. 4.**
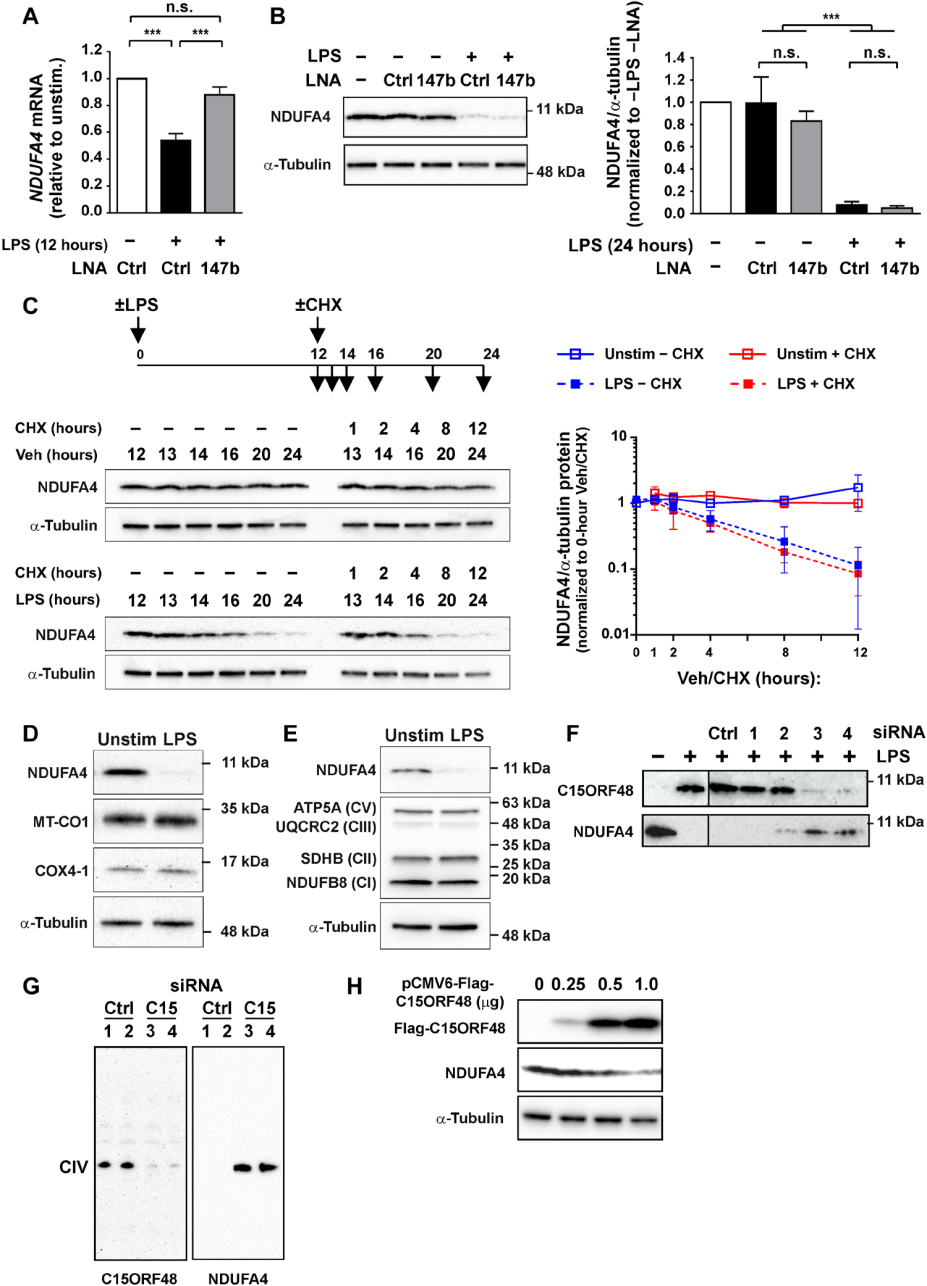
C15ORF48 induces NDUFA4 protein degradation in stimulated macrophages. (**A** and **B**) Human MDMs transfected with control or miR-147b locked nucleic acid (LNA) antisense miRNA antagonist (25 nM), or nontransfected. (A) Cells unstimulated or treated with LPS (10 ng/ml) for 12 hours starting 1 day after transfection. *NDUFA4* mRNA detected by RT-qPCR (*n* = 4; means ± SEM). (B) Cells unstimulated or treated with LPS (10 ng/ml) for 24 hours starting 1 day after transfection. NDUFA4 protein detected by Western blotting. Representative blot of *n* = 3. Graph *n* = 3; means ± SEM. (**C**) CHX chase and Western blotting to determine NDUFA4 protein decay in human MDMs unstimulated [vehicle (Veh)] or treated with LPS (10 ng/ml) for the indicated times with or without addition of CHX (5 μg/ml). Samples collected at time points as indicated by arrows in experimental time line (top). Representative blot (left) and quantification by densitometry normalized to α-tubulin, reported relative to 0 hours CHX (right) (*n* = 3; means ± SD). (**D**) C*c*O subunits detected by Western blotting in human MDMs unstimulated or treated with LPS (10 ng/ml) for 24 hours. Representative blot of *n* = 3. (**E**) Select subunits of ETC complexes detected by Western blotting in human MDMs unstimulated or treated with LPS (10 ng/ml) for 24 hours. Representative blot of *n* = 3. (**F** and **G**) Human MDMs transfected with control or *C15orf48* small interfering RNA (siRNA) (25 nM), or nontransfected. (F) Cells unstimulated or treated with LPS (10 ng/ml) for 24 hours starting 1 day after transfection. C15ORF48 and NDUFA4 proteins detected by Western blotting. Representative blot of *n* = 5. (G) Cells treated with LPS (10 ng/ml) for 24 hours starting 1 day after transfection. BN-PAGE and Western blotting for detection of NDUFA4 and C15ORF48 distribution in ETC complexes and supercomplexes. (**H**) HEK293 cells transfected with the indicated amounts of pCMV6-Flag-C15ORF48 expression plasmid. C15ORF48 (detected with α-Flag antibody) and NDUFA4 proteins detected by Western blotting. Representative blot of *n* = 3. (A and B) One-way ANOVA with Tukey’s correction for multiple comparisons. ***P < 0.001.

To test whether miR-147b is essential for the down-regulation of NDUFA4, we transfected MDMs with a miR-147b–targeting antagomir or nontargeting control, then treated with LPS, and harvested for both mRNA and protein. The miR-147b antagonist prevented the LPS-induced down-regulation of *NDUFA4* mRNA (Fig. 4A) but did not protect NDUFA4 protein (Fig. 4B). We conclude that elevation of miR-147b is sufficient for down-regulation of NDUFA4 protein in transfected MDMs but not necessary for NDUFA4 down-regulation in the context of an LPS stimulus. In addition, note that LPS decreased *NDUFA4* mRNA levels by approximately 50% (Fig. 2C) but decreased NDUFA4 protein levels by greater than 90% (Figs. 3, C and D, and 4, C to F). This suggests that at least one other mechanism may regulate NDUFA4 protein expression at a translational or posttranslational level. To investigate this phenomenon, we treated MDMs with vehicle or LPS for 12 hours (allowing LPS-induced processes to initiate) and then chased those with the protein synthesis inhibitor cycloheximide (CHX) or vehicle for a further 12 hours. LPS caused a steady decrease in NDUFA4 protein levels between the 12- and 24-hour time points (Fig. 4C). The lack of impact of CHX indicates that the phenomenon was not dependent on changes of de novo protein synthesis during this interval. LPS therefore causes active destabilization of NDUFA4 protein.

Under hypoxic conditions, HIF-1α induces expression of *LONP1* and *COX4I2* genes encoding, respectively, the mitochondrial protease LON and the alternative C*c*O subunit COX4-2 (*20*, *22*, *43*). The protease mediates degradation of COX4-1 and permits its substitution by COX4-2, modulating the affinity of the C*c*O complex for oxygen and sustaining its activity. LPS treatment of MDMs decreased the expression of the *LONP1* gene (fig. S2D) and did not alter COX4-1 levels (Fig. 4D). Therefore, despite the involvement of HIF-1α, the response of macrophages to LPS does not resemble a classical hypoxia response, and LON is unlikely to be involved in the degradation of NDUFA4 protein. Unchanged expression of the mitochondrially encoded C*c*O component MT-CO1 (mitochondrially encoded cytochrome ***c*** oxidase 1) (Fig. 4D) and components of other electron transport chain complexes (Fig. 4E) suggests that the degradation of NDUFA4 is selective.

We then tested the effects of four distinct small interfering RNA (siRNA) molecules designed to target *C15orf48* mRNA. Two of the siRNAs effectively blocked the LPS-induced increase in C15ORF48 protein, whereas the other two were nonfunctional (Fig. 4F). Each of the functional siRNAs partially blocked the LPS-induced decrease in NDUFA4 protein. The *C15orf48*-targeting siRNAs also prevented the replacement of NDUFA4 by C15ORF48 in C*c*O in LPS-treated MDMs (Fig. 4G). The differential effects of antagonizing *C15orf48* (Fig. 4, F and G) and miR-147b (Fig. 4B) suggested that expression of C15ORF48 protein itself may contribute to the down-regulation of NDUFA4 protein. To test this hypothesis, we transiently transfected human embryonic kidney (HEK) 293 cells with a vector expressing the C15ORF48 open reading frame but lacking the 3′UTR from which miR-147b is derived. Increasing the quantity of exogenous C15ORF48 protein was sufficient to cause a corresponding down-regulation of endogenous NDUFA4 protein (Fig. 4H). C15ORF48 protein therefore promotes loss of NDUFA4 protein, most likely via its destabilization.

### Expression of *C15orf48* in RA and COVID-19

**Fig. 5.**
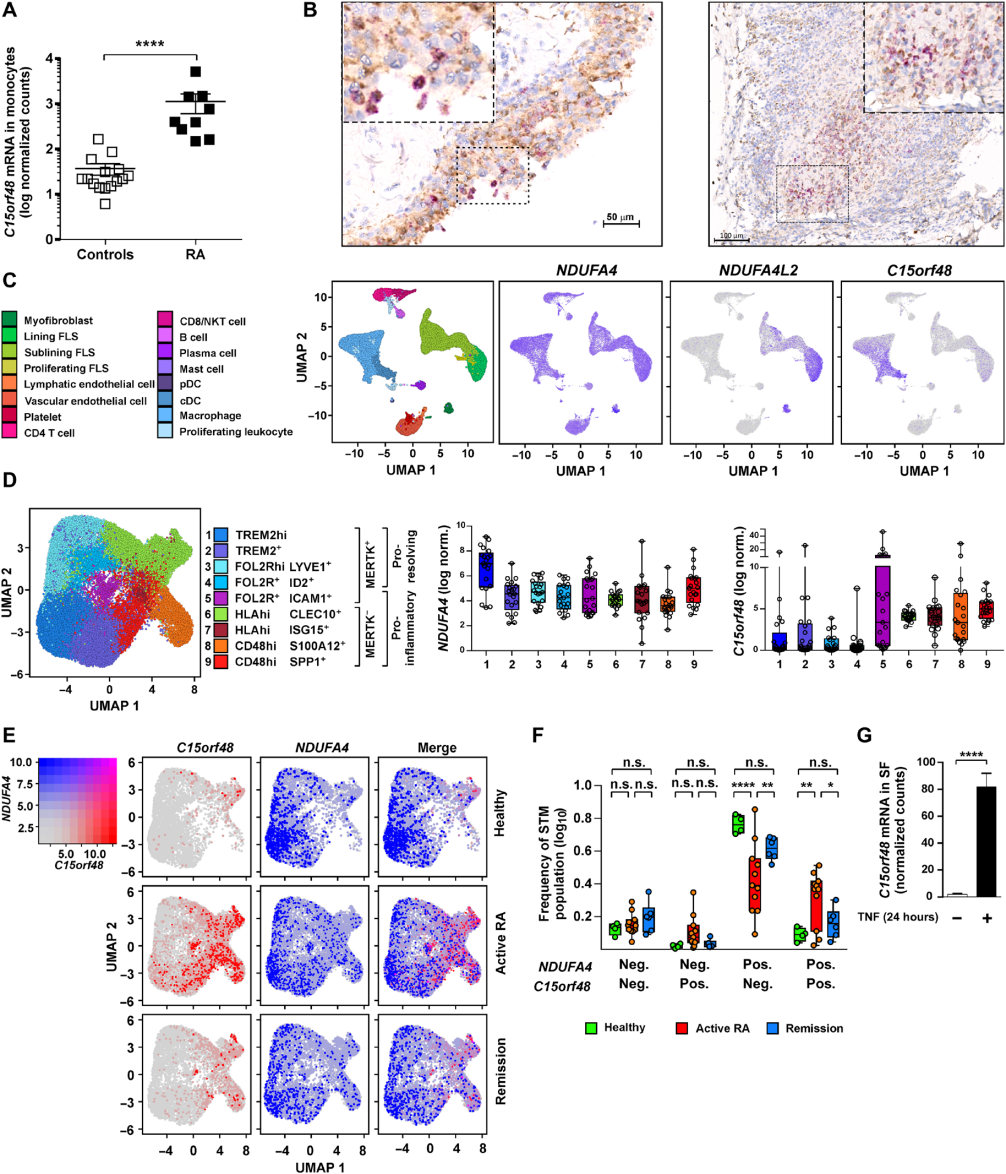
*C15orf48* is expressed during RA and associated with an inflammatory state. (**A**) *C15orf48* expression determined by bulk RNA-seq analysis of peripheral blood CD14^+^ monocytes from healthy controls (*n* = 15) or patients with RA (*n* = 9) (individual data points and means ± SEM are shown). (**B**) *C15orf48* mRNA (pink) detected by RNAscope and CD68 protein (brown) detected by immunohistochemistry in synovial tissue sections from patients with RA undergoing joint replacement. (**C**) UMAP visualization of 37,840 cells from scRNA-seq of synovial tissue of patients with early undifferentiated arthritis, active RA, and RA in remission (nine samples total) (*44*). Left: Cellular identities of identified clusters are indicated. Right: UMAP visualization of *NDUFA4*, *NDUFA4L2*, and *C15orf48* expression across different synovial cell types. The degree of expression is visualized by purple. cDC, conventional dendritic cell; FLS, fibroblast-like synoviocyte; NKT cell, natural killer T cell. pDC, plasmacytoid dendritic cell. (**D** to **F**) scRNA-seq analysis of 32,139 sorted synovial tissue macrophages (STMs) from healthy (*n* = 4), active RA (*n* = 11), and RA in remission (*n* = 6) (*44*). (C) UMAP visualization (left) identifies nine distinct clusters grouped into two main populations based on MerTK expression. Right: Expression of *NDUFA4* and *C15orf48* mRNA in different STM clusters. Data presented as box plots showing median with 25th/75th percentiles and whiskers from minimum to maximum and individual values plotted. Each dot represents an individual healthy donor or patient with RA. (E) UMAP visualization of *C15orf48*^pos^, *NDUFA4*^pos^, and double-positive STMs in healthy, active RA, and RA in remission. (F) Distribution of *C15orf48*^pos^, *NDUFA4*^pos^, and double-positive STMs between conditions. Box plots show median with 25th/75th percentiles and whiskers from minimum to maximum and individual values plotted. Each dot represents individual healthy donors or patient with RA. (**G**) *C15orf48* expression determined by microarray analysis of synovial fibroblasts (SFs) untreated or treated with TNFα in culture for 24 hours (untreated, *n* = 48; TNFα, *n* = 45; means ± SEM). (A and G) Two-tailed *t* test. ****P < 0.0001. (F) Two-way ANOVA with Tukey’s corrections for multiple comparisons.

Bulk RNA sequencing (RNA-seq) was used to analyze differential gene expression in peripheral blood monocytes of patients with RA and healthy controls (K.W. and C.G., manuscript in preparation). Mean *C15orf48* mRNA abundance was 30.48-fold higher in patients with RA than in healthy, age-matched controls (Fig. 5A). This transcript was among the most strongly differentially expressed between patients and controls (*P*_adj_ = 1.1 × 10^−17^).

*C15orf48* expression was elevated in bulk synovial tissue of patients with RA (Table 1). In situ hybridization showed expression of *C15orf48* mRNA almost exclusively in CD68^+^ macrophages of RA biopsies (Fig. 5B), whereas no expression was detected in synovial biopsies of patients with osteoarthritis (OA). Strongly staining cells tended to be found in discrete clusters, which were present in the lining layer (Fig. 5B, left) and inflammatory infiltrates in the sublining layer (Fig. 5B, right). We then used our existing dataset (*44*) to investigate the expression of members of this gene family in the RA synovium at single-cell resolution. *NDUFA4* was broadly expressed in all cell types, whereas expression of *NDUFA4L2* was restricted to myofibroblasts and synovial lining layer fibroblasts, and *C15orf48* mRNA was enriched in cells of myeloid origin (Fig. 5C). These patterns of expression were confirmed in a second, independent single-cell RNA-seq (scRNA-seq) dataset (fig. S3A) (*45*). In this second cohort, expression of *C15orf48* mRNA was significantly elevated in myeloid cells of leukocyte-rich synovial biopsies compared with OA or leukocyte-poor RA biopsies (*P* = 3 × 10^−5^) (fig. S3B). In a third, independent cohort of patients with RA (*46*), synovial biopsies were grouped into three pathotypes on the basis of immunohistology. F (fibroid or pauci-immune) pathotype displays little infiltration of the synovium by immune cells; M (diffuse myeloid) pathotype is characterized by strong myeloid infiltration with little evident cellular organization; L (lympho-myeloid) pathotype is characterized by infiltration of macrophages, T cells, and B cells demonstrating distinct organization into ectopic lymphoid structures. In bulk RNA-seq of synovial biopsies, *C15orf48* mRNA was significantly enriched in biopsies of L and M pathotypes; *NDUFA4L2* mRNA was enriched in M and F pathotypes; and *NDUFA4* displayed no significant differential expression between L-, M-, and F-type synovial biopsies (fig. S4, A and B). Abundance of *C15orf48* mRNA in synovial tissue was significantly positively correlated with measures of disease activity, whereas *NDUFA4* and *NDUFA4L2* displayed no significant correlation with disease activity (fig. S4C).

Our scRNA-seq of synovial biopsies (*44*) identified phenotypic clusters of synovial tissue macrophages (STMs) that broadly fall into two main populations with distinct roles in the regulation of joint inflammation (Fig. 5D, left). MERTK^neg^ (MER tyrosine kinase) STMs promote inflammation by producing cytokines and activating synovial fibroblasts, whereas MERTK^pos^ STMs have broadly anti-inflammatory or proresolution properties and are expanded in resolution of disease. *NDUFA4L2* expression was extremely low in STMs. Expression of *NDUFA4* was quite uniform but highest in population 1 (MERTK^pos^TREM2^high^) macrophages, which are thought to serve a protective, barrier-like function in the healthy joint (Fig. 5D, middle) (*44*, *47*). MERTK^pos^ populations 1 to 4 expressed *C15orf48* weakly, whereas the levels were higher in MERTK^neg^ populations 6 to 9, particularly populations 8 (CD48^high^S100A12^pos^) and 9 (CD48^pos^SPP1^pos^) (Fig. 5D, right). The levels of *C15orf48* mRNA were also relatively high in STMs of population 5 (MERTK^pos^FOL2R^pos^ICAM^pos^), which, uniquely among MERTK^pos^ STMs, display proinflammatory properties (*44*). Increased *C15orf48* expression in proinflammatory STM populations was accompanied by increased expression of several genes involved in aerobic glycolysis and the pentose phosphate pathway (fig. S5) (*8*), both of which fuel proinflammatory functions of myeloid cells (*1*–*10*, *41*). STM expression of *C15orf48* was elevated in active RA but, in remission, returned almost to the levels seen in healthy control synovium (Fig. 5E). The proportion of *NDUFA4^pos^/C15orf48^pos^* macrophages significantly increased in active RA and returned to near healthy levels in remission. Contrarily, the proportion of *NDUFA4^pos^/C15orf48^neg^* macrophages significantly declined in active RA and returned to near healthy levels in remission (Fig. 5F).

Although expression of *C15orf48* mRNA was clearly elevated in STMs, in two independent studies (*44*, *45*), expression was also detected in some synovial fibroblasts (Fig. 5C and fig. S3A). TNF strongly and consistently elevated the expression of *C15orf48* mRNA in synovial fibroblasts (Fig. 5G). Although not conclusive, these observations collectively suggest that increased *C15orf48* expression may contribute to the pathogenesis of RA: *C15orf48* expression is enriched in myeloid cell populations that demonstrate proinflammatory properties; associated with active, myeloid cell–mediated joint inflammation; positively correlated with clinical measures of disease activity; negatively correlated with remission of disease; increased in peripheral monocytes of patients with RA; and up-regulated by a highly disease-relevant proinflammatory stimulus in stromal cells that contribute to joint destruction (*48*).

*C15orf48* expression was also significantly elevated in lung tissue or bronchoalveolar lavage (BAL) of patients with severe COVID-19 (*49*, *50*). We recently reported (*51*) that S100A12^pos^ and SPP1^pos^ STM clusters (clusters 8 and 9 in Fig. 5D), which are abundant in active RA, transcriptionally resemble FCN1^pos^ and FCN1^pos^SPP1^pos^ BAL macrophage clusters that predominate in severe COVID-19 (fig. S6, A and B) (*52*). We therefore analyzed the expression of *C15orf48* and *NDUFA4* in BAL macrophages from healthy controls and patients with COVID-19, using data from Liao and colleagues (*52*). *NDUFA4* expression predominated in resident alveolar macrophages in the healthy state, whereas the FCN1^pos^ and FCN1^pos^SPP1^pos^ alveolar macrophage populations that are enriched in patients with severe COVID-19 and implicated in disease pathogenesis (*51*, *52*) strongly expressed *C15orf48* (fig. S6, C to E). Elevation of *C15orf48* expression is therefore part of the common fingerprint of pathogenic macrophage populations in RA and COVID-19.

### Consequences of remodeling of C*c*O in human macrophages

**Fig. 6.**
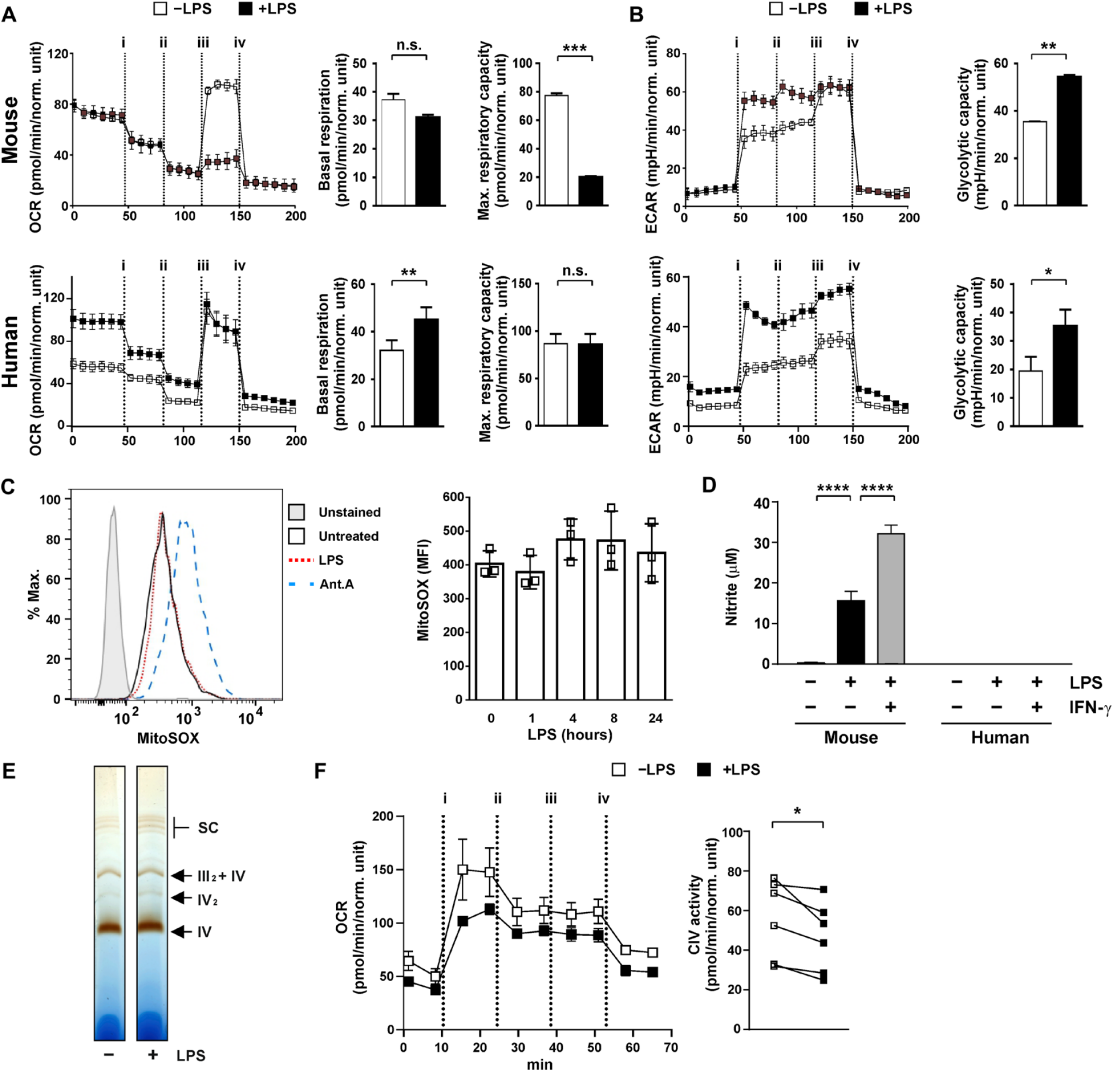
Substitution of NDUFA4 does not influence mitochondrial respiration in macrophages. (**A** and **B**) Seahorse metabolic flux Mito/Glyco stress test of mouse BMDMs and human MDMs unstimulated or treated with LPS (10 ng/ml) for 24 hours. Injections: (i) glucose; (ii) oligomycin; (iii) carbonyl cyanide *p*-trifluoromethoxyphenylhydrazon (FCCP); (iv) rotenone + antimycin A + 2-deoxyglucose. Representative traces and calculated parameters from *n* = 3 (mouse) or *n* = 6 (human) (means ± SEM). ECAR, extracellular acidification rate; OCR, oxygen consumption rate. (**C**) Mitochondrial ROS measurement by MitoSOX staining of human MDMs treated with LPS for 24 hours (left) or the indicated times (right) (*n* = 3, means ± SD). Antimycin A treatment for 1 hour as positive control. (**D**) Nitrite measurement by Griess reaction from conditioned medium of mouse BMDMs and human MDMs treated with LPS (100 ng/ml) ± IFN-γ (100 ng/ml) for 20 hours (*n* = 3 per species, means ± SEM). (**E**) BN-PAGE and in-gel activity assay for complex IV from human MDMs unstimulated or treated with LPS (10 ng/ml) for 24 hours. Representative image of *n* = 3. (**F**) Complex IV activity measurement by Seahorse assay from human MDMs unstimulated or treated with LPS (10 ng/ml) for 24 hours and permeabilized before assaying. Injections: (i) *N*,*N*,*N*′,*N*′-tetramethyl-*p*-phenylenediamine + ascorbic acid; (ii) oligomycin; (iii) rotenone + antimycin A; and (iv) azide. Representative trace (left) and calculated activity from *n* = 6. (A, B, and F) Paired two-tailed *t* test. (C) One-way ANOVA with Dunnett’s correction for multiple comparisons relative to time 0 (no significant differences). (D) Two-way ANOVA with Tukey’s multiple comparisons test. *P < 0.05; **P < 0.01; ***P < 0.001; ****P < 0.0001.

LPS treatment of murine myeloid cells provokes repurposing of mitochondria for production of ROS, accompanied by impairment of oxidative phosphorylation (*40*, *41*). This response is at least partly mediated by nitric oxide as a consequence of up-regulation of *Nos2* gene expression (*6*, *53*). To meet energetic and biosynthetic needs of the cell, aerobic glycolysis is increased in parallel. We hypothesized that the substitution of NDUFA4 by C15ORF48 contributes to these profound metabolic changes. However, the LPS responses of human macrophages were found to differ from those of murine macrophages. LPS treatment of mouse BMDMs caused the expected impairment of maximum mitochondrial respiratory capacity but no change in basal respiration (Fig. 6A, top). In contrast, LPS increased basal respiration of human macrophages but did not affect maximal respiratory capacity (Fig. 6A, bottom). Glycolytic capacity was increased by LPS in macrophages of both species (Fig. 6B), indicating that Warburg-like metabolism can be uncoupled from impairment of oxidative phosphorylation. LPS treatment of human macrophages did not significantly increase mitochondrial ROS production (Fig. 6C). Because the switch between NDUFA4 and C15ORF48 occurs in LPS-treated mouse and human macrophages but human macrophages do not lose respiratory capacity under these conditions, we suggest that the subunit exchange within C*c*O is not responsible for impaired oxidative phosphorylation. The fact that repurposing of mitochondria for ROS production does not occur in human MDMs may be explained by the fact that, unlike murine BMDMs, they do not produce nitric oxide in response to LPS, even when IFN-γ is added (Fig. 6D).

We directly measured C*c*O activity in macrophages to gain better insight into the potential consequences of the subunit switch. In-gel activity assays did not show prominent changes of C*c*O activity in response to LPS (Fig. 6E). A more quantitative complex-specific metabolic flux assay revealed a small but statistically significant (16 ± 4%) LPS-induced reduction of C*c*O activity (Fig. 6F). We attempted to use RNA interference to investigate the metabolic and functional consequences of remodeling of the C*c*O complex. However, the process of transfecting primary MDMs (using three chemically distinct transfection reagents) altered both metabolic functions and inflammatory outputs. Three different control siRNAs exerted additional donor- and gene-specific effects, rendering conclusions about specific consequences of NDUFA4 or C15ORF48 knockdown impossible. Electroporation also caused sustained mitochondrial ROS production indicating disruption of metabolism. Derangement of cellular metabolism is a hallmark of oncogenic transformation (*54*); for example, LPS did not increase aerobic glycolysis in the mouse macrophage cell line RAW264.7. We reasoned that knockdown or gene editing approaches in transformed myeloid cell lines were likely to generate misleading results.

**Fig. 7.**
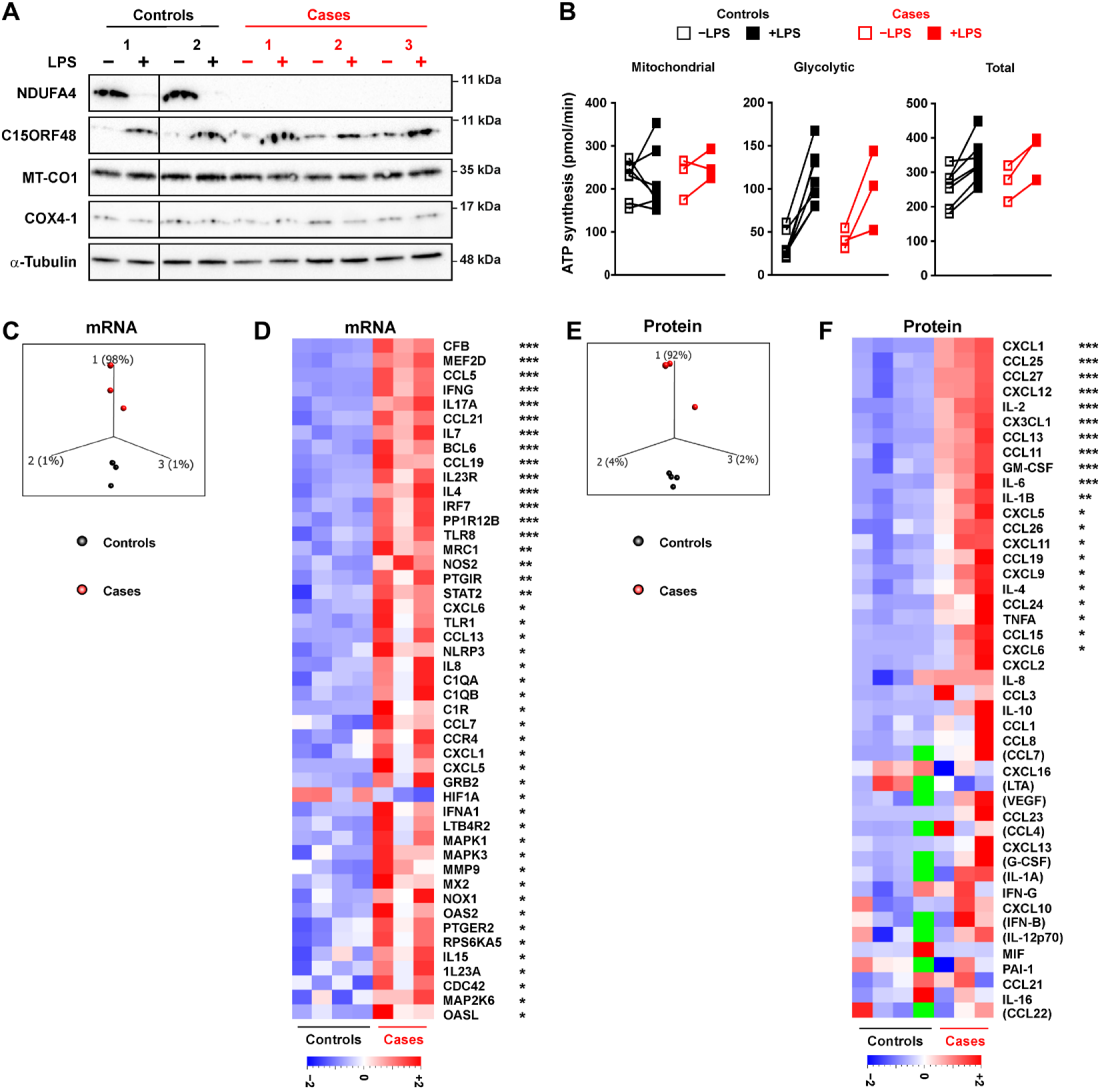
Human MDMs lacking NDUFA4 protein show a hyperinflammatory phenotype. (**A**) Western blot of human MDMs from NDUFA4-null patients (cases) or healthy controls unstimulated or treated with LPS (10 ng/ml) for 24 hours. (**B**) ATP synthesis rate measured by Seahorse assay of human MDMs from NDUFA4-null patients (cases) or healthy controls unstimulated or treated with LPS (10 ng/ml) for 24 hours (controls, *n* = 7; cases, *n* = 3). The NanoString Human Inflammation panel was used to examine gene expression in resting macrophages of cases and controls. (**C**) Principal components analysis. (**D**) Heatmap of all genes significantly differently expressed between controls and cases. Two multiplexed bead immunoassays were used to measure secreted factors in supernatants of resting macrophages from NDUFA4-null cases and healthy controls. (**E**) Principal components analysis. (**F**) Heatmap of all analytes that were expressed at detectable levels. Green blocks indicate missing values (three control supernatants were used in the second multiplexed assay). (B) Two-way ANOVA with Sidak correction for multiple comparisons (no significant difference between groups). (E and F) ANOVA performed to identify significantly differentially expressed genes within whole datasets. *P < 0.05; **P < 0.01; ***P < 0.001.

We therefore turned our attention to a very rare genetic condition, in which a mutated allele of the NDUFA4 gene produces no detectable NDUFA4 protein (*26*). Individuals who are homozygous for this loss-of-function mutation display severe neuromuscular symptoms, but inflammatory features have not previously been investigated. Monocytes were isolated from all three known, surviving homozygous individuals, and differentiated into macrophages in vitro. In these macrophages, there was a de facto substitution of NDUFA4 by C15ORF48 under resting conditions (Fig. 7A). Basal expression of C15ORF48 was possibly increased in the absence of NDUFA4, but the limited number of replicates makes this conclusion tentative at best. The levels of the C*c*O components MT-CO1 and COX4-1 were unaffected by the NDUFA4 mutation. These macrophages represent a model for the specific substitution of NDUFA4 in the absence of a proinflammatory stimulus. Under resting conditions or following stimulation with LPS, there were no significant differences between NDUFA4-null macrophages and those derived from healthy controls in terms of mitochondrial, glycolytic, or total adenosine 5′-triphosphate (ATP) production rates (Fig. 7B). Constitutive loss of NDUFA4 therefore did not seem to impair mitochondrial ATP production or cause a shift to glycolytic metabolism under resting conditions in this cell type; neither did it influence the metabolic response to LPS.

Gene expression was compared between control and NDUFA4-null macrophages using a NanoString inflammation panel. We focused on unstimulated macrophages because differences in expression of C*c*O components were most notable under this condition (Fig. 7A). Principal components analysis clearly separated NDUFA4-null from control macrophages, with the first principal component accounting for 98% of experimental variance (Fig. 7C). Controls clustered together well, whereas cases were more dispersed, suggesting that there may be variations in penetrance of the condition caused by loss of NDUFA4 function. Across 249 inflammation-related transcripts represented in the panel, 47 were significantly (*P* < 0.05) differentially expressed between NDUFA4-null and control macrophages (Fig. 7D). All were overexpressed by NDUFA4-null macrophages with the single exception of *HIF1A* (fold difference case/control = 0.652, *P* = 0.026). One of the most notable differentially expressed genes was *CFB*, encoding complement factor B, the C3 convertase of the alternative pathway (fold difference case/control = 16.0, *P* = 0.00018). Expression of the complement genes *C1QA*, *C1QB*, and *C1R* was also elevated in NDUFA4-null macrophages (Fig. 7D), whereas other complement genes were either not expressed or not differentially expressed.

Several genes encoding cytokines and CCL and CXCL chemokines were overexpressed at the mRNA level by unstimulated NDUFA4-null macrophages (Fig. 7D). To confirm this broadly proinflammatory pattern, we used multiplexed bead immunoassays to quantify secreted factors in supernatants of unstimulated control and NDUFA4-null macrophages. Principal components analysis again demonstrated clear separation between control and NDUFA4-null macrophages, 92% of experimental variance being accounted for by the first principal component (Fig. 7E). Again controls clustered well, whereas cases were more dispersed in PC1. Twenty-one of 45 analytes were significantly overexpressed by NDUFA4-null macrophages, whereas none were significantly underexpressed (Fig. 7F; absolute quantities are plotted in fig. S7). The expression of several cytokines and chemokines was increased by more than 10-fold in NDUFA4-null macrophages (fig. S7), for example, CCL13 (10.75-fold), CXCL1 (12.78-fold), and CXCL2 (13.58-fold). In some cases, fold differences of expression had to be estimated because levels were either below detection limit in control or above detection limit in NDUFA4-null macrophages. For example, IL-6 was overexpressed at least 40-fold by NDUFA4-null macrophages (fig. S7). Several secreted factors (for example, CCL21 and IL-12p70) were well within linear range of the assay and did not differ significantly between control and NDUFA4-null macrophages, emphasizing that constitutive loss of NDUFA4 has broad but not indiscriminate effects on macrophage biology. Although the NanoString and multiplexed bead panels did not perfectly overlap, where they did overlap there was generally good agreement as to altered expression at mRNA and protein levels. Collectively, these data reveal a notable proinflammatory phenotype of macrophages lacking NDUFA4 protein, suggesting that exchange of C*c*O components plays an important role in the regulation of macrophage functions.

## DISCUSSION

C15ORF48 (NMES1) was originally described as a nuclear protein (*36*); however, this was based on low-resolution immunohistology using an unvalidated polyclonal antiserum. Although lacking canonical mitochondrial localization signals, both NDUFA4 and C15ORF48 are now annotated as mitochondrial proteins in the expert-curated databases MitoCarta (*55*) and the Integrated Mitochondrial Protein Index (www.mrc-mbu.cam.ac.uk/impi). This annotation is supported by published evidence (*26*, *38*, *55*–*57*) including confocal microscopy and cell fractionation experiments using overexpressed C15ORF48 (*38*, *56*). To this, we have added cell fractionation of primary human macrophages and Western blotting of endogenous proteins with antibodies validated by knockdown, showing predominantly mitochondrial staining of both proteins (Fig. 3G). Under conditions of maximal expression (without and with LPS in the cases of NDUFA4 and C15ORF48, respectively), we could detect very weak staining of each protein in nuclear fractions. This was almost certainly a result of mitochondrial contamination, because similar weak staining of the mitochondrial protein SDHA (succinate dehydrogenase complex subunit A) could be detected in nuclear fractions. We conclude that both NDUFA4 and C15ORF48 are exclusively, or almost exclusively, mitochondrial proteins.

The *NDUFA4* and *C15orf48* genes share an extraordinarily complex relationship. They have limited sequence similarity but their exon-intron structures are the same. The positions of the first and third exon boundaries are identical, while the second is shifted by only two nucleotides, a very high degree of relatedness that points to an origin in gene duplication. The encoded proteins share less than 30% amino acid identity but are predicted to have similar secondary structures and electrostatic properties (Fig. 3B and fig. S1, A and B) (*56*, *57*). The 3′UTR of *C15orf48* mRNA is also the source of the miRNA miR-147b. The protein and miRNA products of the *C15orf48* transcript converge to bring about down-regulation of NDUFA4 protein levels; miR-147b specifically recognizes a conserved site within the *NDUFA4* 3′UTR, causing down-regulation of this target mRNA (Fig. 2); and C15ORF48 protein promotes loss of NDUFA4 protein (Fig. 4H), most likely by inducing its degradation (Fig. 4C). While this manuscript was in preparation, other researchers described similar combinatorial effects of miR-147b and C15ORF48 protein on NDUFA4 expression in transfected HEK293 (embryonic kidney) or A549 (airway epithelial) cell lines (*56*, *57*). The exceptional evolutionary conservation of both protein- and miRNA-encoding portions of the *C15orf48* gene (Fig. 1A) suggests that this complex phenomenon has been subject to powerful positive selection for some hundreds of millions of years (*56*).

Not all of the effects of miR-147b can be attributed to down-regulation of NDUFA4 (*57*). miRNAs commonly target several transcripts within a single biological process or pathway; therefore, we were interested in two putative miR-147b targets that encode mitochondrial proteins; *SDHD* and *ALDH5A1* (aldehyde dehydrogenase 5 family member A1) (*58*, *59*). miR-147b–mediated down-regulation of *SDHD* was reported to contribute to TCA cycle dysfunction in lung adenocarcinomas (*59*). The predicted interactions between miR-147b and *SDHD* mRNA were not conserved in the mouse. Furthermore, *SDHD* mRNA levels in MDMs were not significantly reduced by either LPS treatment or transfection of a miR-147b mimic. Seed sequence interactions between miR-147b and *ALDH5A1*/*Aldh5a1* mRNAs were conserved between human and mouse, but there were few base pairs outside the seed region. *ALDH5A1* mRNA was strongly down-regulated following treatment of human MDMs with LPS, but transfection with a miR-147b mimic failed to recapitulate this effect. We conclude that neither *ALDH5A1* nor *SDHD* is likely to be a major target of miR-147b in MDMs and that further relevant targets remain to be identified.

As indicated by its name, NDUFA4 was originally thought to be a component of mitochondrial electron transport chain complex I (the NADH dehydrogenase complex). More recent cryo-EM structural evidence places NDUFA4 at the outer surface of the C*c*O complex (complex IV) (*28*), and molecular modeling suggests that C15ORF48 could occupy the same position (Fig. 3B). Consistent with these reports, in primary human macrophages, the pattern shown by NDUFA4 and C15ORF48 proteins was consistently that of C*c*O complex subunits (Figs. 3H and 4G). Others have reported similar findings (*25*, *26*, *57*, *60*), lending strong support to a change in systematic name from *NDUFA4* to *COXFA4* (*27*). LPS treatment of primary human macrophages caused an increase in levels of C15ORF48 protein and a reciprocal decrease in levels of NDUFA4 (Fig. 3C), resulting in substitution of NDUFA4 by C15ORF48 in all C*c*O complexes (Fig. 3H). LPS-induced degradation of NDUFA4 protein (Fig. 4C) may be a consequence of its exclusion from C*c*O (*57*). Overexpression of C15ORF48 caused a similar protein substitution in C*c*O complexes of mouse heart mitochondria (*57*). Efficient substitution of NDUFA4 by C15ORF48 in C*c*O also occurred during mouse spermatogenesis (*60*), most likely involving the mechanisms described above. The question is why cells should go to such lengths to replace one mitochondrial protein with another. Put another way, what are the consequences of the replacement of NDUFA4 by C15ORF48?

Depletion of NDUFA4 impaired C*c*O function in HeLa or muscle cells (*25*, *26*). HeLa cells do not express *C15orf48* mRNA in either normoxia or hypoxia, and muscle tissue expresses little or no C15ORF48 protein (Human Protein Atlas); therefore, the decrease in C*c*O activity upon NDUFA4 depletion in these studies likely represents conditions in which neither protein is present. In contrast, we found only very slight impairment of C*c*O function in LPS-treated MDMs (Fig. 6F), and no deficit in mitochondrially derived ATP production due to either genetically determined or LPS-induced loss of NDUFA4 protein (Fig. 7B). Therefore, NDUFA4 is dispensable for mitochondrial respiration in MDMs, most likely because it can be functionally replaced by C15ORF48, even under resting conditions (Fig. 7, A and B). Under hypoxic conditions, HIF-1α promotes the substitution of NDUFA4 by NDUFA4L2, mitigating mitochondrial ROS production and consequent cell death (*30*, *32*, *34*, *42*). Others have reported or speculated that substitution of NDUFA4 by C15ORF48 similarly reduces mitochondrial ROS production under hypoxia or other stressful conditions (*56*, *57*, *60*). This appears not to be the case in primary human MDMs, for the following reasons: (i) Although LPS activates HIF-1α and decreases NDUFA4 protein levels in MDMs, these occur in the absence of any changes of C15ORF48 expression (fig. S2). (ii) Unlike mouse BMDMs, human MDMs do not produce nitric oxide (Fig. 6D), increase mitochondrial ROS production (Fig. 6C), or display impairment of oxidative phosphorylation (Fig. 6A) in response to LPS. (iii) In mouse myeloid cells, LPS-induced mitochondrial ROS production is principally mediated by reverse electron transport at complex I (*61*). There is no documented mechanism by which alterations in composition of C*c*O (complex IV) can influence this process. In the absence of new evidence, consequences of the NDUFA4-C15ORF48 substitution must be sought elsewhere.

An alternative hypothesis is that exchange of subunits affects the ability of C*c*O to participate in higher-order electron transport chain structures. Dimerization of C*c*O was proposed to exclude the binding of NDUFA4, with effects on respiratory efficiency and sensitivity to negative feedback by high intracellular concentrations of ATP (*21*). However, both NDUFA4 and C15ORF48 were detected in both C*c*O monomeric and dimeric structures (Fig. 3H), implying that the switch does not contribute to regulation of dimerization. Both NDUFA4 and C15ORF48 can interact with subunits of complexes I and III, leading to the suggestion that they may regulate the formation of higher-order electron transport chain complexes (*38*, *62*, *63*). Assembly factors by definition do not stably associate with the complexes that they help to form. Both NDUFA4 and C15ORF48 could be consistently detected in supercomplexes (Fig. 3H) (*28*), and therefore, they do not seem to be supercomplex assembly factors but rather structural components of C*c*O necessary for its optimal activity (*26*). We reproducibly observed an increase in abundance of supercomplexes following LPS treatment of MDMs (fold increase 1.90 ± 0.24 SEM; *n* = 4; *P* < 0.01) (Fig. 6E). However, we cannot yet conclude whether this increase was dependent on exchange of C*c*O subunits. We also cannot rule out functions that are not directly related to oxidative phosphorylation. For example, loss of NDUFA4 has been linked to increased sensitivity to stress-induced apoptosis (*56*). Functions outside of the mitochondria appear unlikely.

Expression of *C15orf48* was increased by LPS treatment of primary macrophages from mouse, human (Fig. 1) (*37*), and several other mammalian species (*64*). It was also increased by treatment of synovial fibroblasts with TNF (Fig. 5G); A549 cells (*56*) or mouse macrophages with type I IFN (Fig. 1G); RAW264.7, A549, HL-60, and L929 cells with type II IFN (*56*); A549 cells or astrocytes with IL-1β (*65*, *66*); microglia with M1 polarizing agonists LPS and/or IFN-γ (*67*); and natural killer cells with IL-2 and IL-12 (*68*). Of course, these potent responses to proinflammatory agonists do not prove a proinflammatory role for *C15orf48*. Most reports to date, using overexpression in a variety of primary and transformed cell types, agree that the protein and miRNA products of *C15orf48* mRNA have anti-inflammatory effects (*37*, *57*, *66*, *69*, *70*). However, a proinflammatory function of miR-147b was previously reported (*71*) and is also suggested by the studies described here.

Expression of *C15orf48* was notably increased under several inflammatory conditions of autoimmune, metabolic, cardiovascular, bacterial, or viral nature (Table 1). These associative data still do not prove that *C15orf48* has a causative role in inflammatory pathologies; therefore, we investigated in more detail the expression of *C15orf48* and its relatives in RA. Expression of *C15orf48* was positively correlated with disease activity scores in bulk RNA-seq of RA synovial biopsies (fig. S4) and decreased in remission of disease (Fig. 5E). By scRNA-seq *C15orf48* expression was found to be strongly enriched in STM populations having demonstrable proinflammatory functions (Fig. 5D) (*44*). In these cell populations, elevated expression of *C15orf48* was accompanied by elevated expression of genes belonging to glycolytic and pentose phosphate pathways (fig. S5) (*8*, *44*). *NDUFA4* was expressed quite widely but most strongly in TREM2^high^ STMs (Fig. 5D), which are believed to play an important role in the preservation of synovial joint integrity (*44*, *47*). In this population, elevated expression of *NDUFA4* was accompanied by elevated expression of genes belonging to the oxidative phosphorylation pathway and by low expression of genes contributing to aerobic glycolysis and the pentose phosphate pathway (fig. S5) (*8*, *44*).

Analogous to its up-regulation in proinflammatory synovial macrophages during RA, *C15orf48* was also found to be elevated in severe COVID-19 and to be expressed by subsets of macrophages that have been implicated in pathogenic processes (fig. S6) (*51*, *52*). In addition, mirroring RA STM subsets, *NDUFA4*-expressing alveolar macrophages (FABP4^pos^) showed elevated expression of genes related to oxidative phosphorylation and fatty acid metabolism and decreased glycolytic gene expression, while the opposite was true for *C15orf48*-expressing subsets (FCN1^pos^ or FCN1^pos^SPP1^pos^) (*52*). Divergent expression of *NDUFA4* and *C15orf48* in macrophage populations therefore appears to be part of a consistent pattern of metabolic commitment to either homeostatic or proinflammatory, destructive processes across different diseases (*72*). Mitochondrial dysregulation has been linked to pathogenic processes in RA (*8*), and in COVID-19, mitochondrial fitness has been posited as a determinant of COVID-19 disease severity (*73*). Severe acute respiratory syndrome coronavirus 2 viral components have also been suggested to interact with mitochondria to facilitate viral replication and evade host immunity, thereby disrupting mitochondrial function (*73*–*75*). These hypotheses still require full experimental validation, and it will be interesting to ascertain the precise role that the *C15orf48/NDUFA4* axis plays in these disease processes.

Mutations of the *NDUFA4* gene allowed us to ask what happens in primary human macrophages that already lack NDUFA4 protein in the absence of any stimulus, and in which only C15ORF48 protein is available to occupy the position on the outer surface of C*c*O. Under resting conditions, these macrophages very strongly overexpressed a large number of secreted factors, including several chemokines of the CCL and CXCL families (Fig. 7 and fig. S7). It remains to be determined whether dysregulated innate immunity contributes to the severe condition caused by homozygous *NDUFA4* mutations (*26*). Collectively, these observations provide circumstantial evidence that the up-regulation of *C15orf48* mRNA and the consequent replacement of NDUFA4 by C15ORF48 in C*c*O promote inflammation, although there are contradictory data derived from in vitro experiments. Conclusive evidence may depend on the development of better experimental tools, such as conditional knockout mouse strains. The notable evolutionary conservation of the mechanism and its recapitulation at many sites of inflammatory pathology suggest that further research is strongly justified.

## MATERIALS AND METHODS

### Macrophage isolation and culture

Human monocytes from healthy blood donors were isolated from leukapheresis blood cones supplied by the National Blood and Transplant Service (ethical approval ERN_16-0191). Monocytes were enriched by negative selection using STEMCELL RosetteSep Human Monocyte Enrichment Cocktail (STEMCELL Technologies. 15068; blood, 75 μl/ml) and Ficoll-Paque (VWR, 17144003). Cells were differentiated for 7 days in RPMI 1640 medium with l-glutamine (Gibco, Thermo Fisher Scientific, 21875034) supplemented with 5% heat-inactivated fetal bovine serum (Biosera, FB-1001) and recombinant macrophage colony-stimulating factor (M-CSF) (50 ng/ml; PeproTech, 300-25).

All mice were maintained at the Biomedical Services Unit of the University of Birmingham. Animal care and experimental procedures were performed according to the Home Office guidelines and approved by the University of Birmingham Local Ethical Review Committee. Wild-type (WT) C57/BL6J mice between 6 and 12 weeks of age were humanely culled, bone marrow–flushed from femurs, and BMDM-obtained by culture for 7 days in RPMI 1640 with l-glutamine (Gibco, Thermo Fisher Scientific, 21875034) supplemented with 10% heat-inactivated fetal bovine serum (Sigma-Aldrich, F0392) and recombinant M-CSF (50 ng/ml; PeproTech, 300-25).

Stimulations were carried out in 12-well culture plates at 0.5 × 10^6^ cells per well (human) or 1 × 10^6^ cells per well (mouse) or in 6-well culture plates at 1 × 10^6^ cells per well (human) or 2.5 × 10^6^ cells per well (mouse) using the following reagents and concentrations, unless otherwise stated: LPS (10 ng/ml; Enzo, ALX-581-010-L002), recombinant human IFN-β (10 ng/ml; PeproTech, 300-02 BC—cross-reacts with mouse), Rux (1 μM; Selleck, S1378), CHX (5 μg/ml; Sigma-Aldrich, 01810), human TLR agonist kit (Invivogen, tlrl-kit1hw), Pam3CSK4 (1 μg/ml; TLR1/2), heat-killed *Listeria monocytogenes* (10^8^ cells/ml; TLR2), poly(I:C) high molecular weight (10 μg/ml; TLR3), poly(I:C) low molecular weight (10 μg/ml; TLR3), flagellin from *Salmonella typhimurium* (100 ng/ml; TLR5), FSL-1 (diacylated lipopeptide Pam2CGDPKHPKSF) (1 μg/ml; TLR6/2), imiquimod (1 μg/ml; TLR7), ssRNA40 (10 μg/ml; TLR8), and ODN2006 (5 μM; TLR9). All stimulations were carried out in the absence of M-CSF.

### Studies of patients with RA

#### Monocytes

Peripheral blood was collected from patients with RA with active disease (DAS28 > 2.8), Gartnavel General Hospital Rheumatology, Glasgow. Healthy controls were obtained from age-matched volunteers at the University of Glasgow. Informed consent was obtained before sample collection alongside the appropriate ethical approval (West of Scotland REC 4, approval 19/WS/0111, project reference CG_2019_09_A_AM01).

Peripheral blood mononuclear cells (PBMCs) were separated by Ficoll-Paque density centrifugation. CD14^+^ monocytes were magnetically isolated from PBMCs using the EasySep Human CD14 Positive Selection Kit II (STEMCELL Technologies). Isolated CD14^+^ monocytes were lysed using the RNA Lysis Buffer containing β-mercaptoethanol, and RNA was extracted using the PureLink RNA Mini Kit (Invitrogen). RNA samples were sequenced by GenomeScan BV (Leiden, The Netherlands). RNA sample libraries were prepared using the NEBNext Ultra II Directional RNA Library Prep Kit for Illumina [New England BioLabs (NEB)]. RNA-seq was performed using the NovaSeq 6000 (Illumina), paired-end 150 base pairs, 20 million reads, using 1.1 nM DNA. The reads generated by RNA-seq were aligned to the reference genome (GRCh38.p13, Ensembl) using STAR (version 2.7.3), with default settings. DESeq2 was then used to normalize read counts and identify differentially expressed genes between sample groups.

#### scRNA-seq data

Participants fulfilling the American College of Rheumatology 2010 revised criteria for RA were recruited and underwent ultrasound-guided synovial tissue biopsy of the knee at the Division of Rheumatology of Fondazione Policlinico Universitario A. Gemelli IRCCS, Università Cattolica del Sacro Cuore, Rome, Italy. The work was approved by the Ethics Committee of the Università Cattolica del Sacro Cuore (no. 6334/15) and by the West of Scotland Research Ethics Committee (no. 19/WS/0111). All participants provided signed informed consent. Clinical and demographic information, sample preparation, scRNA-seq data generation, and data analysis are described (*51*). “Healthy” group comprises healthy donors attending arthroscopy for meniscal tear or cruciate ligament damage and with normal synovium (via magnetic resonance imaging and macroscopically) (University of Glasgow). “Active RA” group includes treatment-naïve patients and treatment-resistant patients (inadequate responder to methotrexate). “Remission” group comprises participants in sustained (minimum of 6 months) clinical and ultrasound remission under methotrexate and TNF inhibitor therapy.

#### Synovial fibroblasts

Synovial tissue samples were obtained from patients undergoing joint replacement surgery or by ultrasound-guided biopsy from treatment-naïve patients attending an early arthritis clinic with joint pain and/or inflammation of duration of less than 12 weeks . Fibroblasts were isolated as previously described (*76*). Between passages 4 and 6, fibroblasts were left untreated or stimulated with TNF (100 ng/ml) for 24 hours. Differential gene expression was determined by Agilent microarray (A.F. in preparation). Stratification of patients (*76*) revealed no differences in the response of the *C15orf48* gene to TNF according to disease stage or outcome; therefore, all 48 independent synovial fibroblast isolates were grouped together. All human samples were obtained with written, informed consent and approval from the West Midlands Black Country Local Research Ethics Committee (references 07/H1204/191 and 07/H1203/57).

### NDUFA4 patients

Three individuals suffering from NDUFA4 mutation-associated Leigh syndrome were recruited at the Queen Square Centre for Neuromuscular Diseases, University College London, under the ethical approval by the Queen Square Research Ethics Committee, London (no. 09/H0716/76). Informed consent was obtained from all participants. Genetic and clinical patient details are described in (*26*). Monocytes were isolated from peripheral blood and differentiated into macrophages as described above.

### Reverse transcription quantitative polymerase chain reaction

RNA was isolated using the Norgen Total RNA Purification Plus Kit (Geneflow, P4-0016) according to the manufacturer’s instructions. cDNA was synthesized to allow miRNA and mRNA detection using the miScript II RT Kit (QIAGEN, 218161). mRNA was detected by reverse transcription quantitative polymerase chain reaction (RT-qPCR) using SYBR TB Green Premix Ex Taq (Takara, RR820W) and primers supplied by Eurofins Genomics (Table 2). UBC (human) or Rpl13a (mouse) were used to normalize mRNA measurements via 2^−ΔΔ*C*t^ method.

**Table 2.** RT-qPCR primer sequences.

**Target gene**	**Species**	**Forward primer**	**Reverse primer**
*C15orf48*	Human	AACTCATTCCCTTGGTGGTGTTCAT	CTCGTCATTTGGTCACCCTTTGGAC
AA467197 (*Nmes1*/*C15orf48*)	Mouse	AGGAACTCATTCCTTTGGCGT	TTTTCCGATCAATAACCACGTC
*NDUFA4*	Human	AAGCATCCGAGCTTGATCCC	ACAATGCCAGACGCAAGAGA
*NDUFA4L2* (primer set 1)	Human	TTCTACCGGCAGATCAAAAGACA	GGGCGAGTCGCAGCAA
*NDUFA4L2* (primer set 2)	Human	CAAAAGACATCCGGGGATCA	GCGAGTCGCAGCAAGTAAAG
*LONP1*	Human	CGGGAAGATCATCCAGTGTT	ACGTCCAGGTAGTGGTCCAG
*UBC*	Human	CGGGATTTGGGTCGCAGTTCTTG	CGATGGTGTCACTGGGCTCAAC
*Rpl13a*	Mouse	GCGGATGAATACCAACCCCT	CCACCATCCGCTTTTTCTTGT

miRNAs were detected by RT-qPCR using miScript SYBR Green (QIAGEN, 218075) and Hs_miR-147b_1 or Mm_miR-147_2 miScript Primer Assays (QIAGEN). Hs_RNU6-2_11 miScript Primer Assay (QIAGEN) was used to normalize miRNA measurements via 2^−ΔΔ*C*t^ method.

### miRNA target analysis

Target prediction for hsa–miR-147b-3p was performed using TargetScan7.1, miRTarBase, miRDB, and miRanda (microRNA.org). The results were compared for commonly predicted targets. Target match between mmu-miR-147-3p and mmu-*Ndufa4* was analyzed using miRanda (microRNA.org).

### Transfections

Macrophage transfections were performed on day 6 of macrophage differentiation in 12-well culture plates using Mirus TransIT-X2 reagent (Geneflow, E7-0106) according to the manufacturer’s instructions. miRNA mimic experiments were performed using Dharmacon miRIDIAN human miR-147b or negative control mimics (2 nM; Horizon Discovery). Antagomir experiments were performed using miRCURY LNA (locked nucleic acid) human miR-147b or negative control power inhibitors (25 nM; QIAGEN). siRNA transfections were performed using Dharmacon siGenome siRNAs and nontargeting controls (25 nM; Horizon Discovery). Nontransfected samples were treated with TransIT-X2 transfection reagent alone. Samples were harvested 24 hours after transfection for RNA analysis or 48 hours after transfection for protein analysis.

HEK293 transfections for Western blot were performed in six-well culture plates using Mirus TransIT-X2 reagent (Geneflow, E7-0106) according to the manufacturer’s instructions. C15ORF48-MycDDK expression plasmid was obtained from OriGene.

### Western blotting

For whole-cell lysates, cells were harvested in radioimmunoprecipitation assay buffer and samples passed through a QIAshredder column to remove genomic DNA (QIAGEN, 79656). Cell fractionation was performed essentially as described in (*77*), using 10 × 10^6^ to 20 × 10^6^ primary MDMs per condition and a 2-ml Dounce tissue grinder with small clearance pestle (30 strokes) (Sigma-Aldrich, D8938). Nuclear fraction was taken as pellet following homogenization and centrifugation at 1200*g*. Cytoplasmic fraction was taken as supernatant following mitochondrial sedimentation at 17,000*g*. Protein was quantified by the Pierce BCA Assay (Thermo Fisher Scientific, 23225). Laemmli buffer was added and samples heated to 95°C for 5 min (unless blotting for MT-CO1). Western blotting was performed using Criterion TGX protein gels (Bio-Rad) and tris-glycine SDS buffer (Geneflow, B9-0032) or using XT bis-tris protein gels (Bio-Rad) and XT MES running buffer (Bio-Rad, 1610789). Protein was transferred to Bio-Rad Trans-Blot polyvinylidene difluoride (PVDF) membranes (Bio-Rad, 1704157) using Bio-Rad Trans-Blot Turbo transfer system. The following antibodies and dilutions were used: C15orf48 (1:1000; Antibodies-online.com, ABIN2784037); NDUFA4 (1:5000; Thermo Fisher Scientific, PA5-50068); α-tubulin (1:2000; Sigma-Aldrich, T9026); SDHA (1:2000; Abcam, ab14715); lamin A/C (1:1000; BD Biosciences, 612163); MT-CO1 (1:1000; Abcam, ab14705); COX4-1 (1:1000; Abcam ab14744); total oxidative phosphorylation human WB antibody cocktail (1:500; Abcam, ab110411); anti-rabbit immunoglobulin G (IgG), horseradish peroxidase (HRP)–linked (1:2000; NEB, 7074SNEB); and anti-mouse IgG, HRP-linked (1:2000; NEB, 7076SNEB). Blots were imaged using Clarity Western ECL Substrate (Bio-Rad, 1705061) and Bio-Rad ChemiDoc MP Imaging System. Densitometry for Western blot quantification was performed using ImageJ.

### Luciferase 3′UTR reporter assay

WT NDUFA4 3′UTR was amplified from human genomic DNA and cloned into the pmirGLO Dual-Luciferase miRNA Target Expression Vector (Promega, E1330) using standard molecular cloning methods. ImiRP computational biology tool (imirp.org) was used to predict tolerated mutation of the miR-147b target site without the generation of new miRNA binding sites. The WT miR-147b seed sequence target site (CCGCACA) was mutated to CCGCGGG to produce pmirGLO NDUFA4mut using the QuikChange II Site-Directed Mutagenesis Kit (Agilent, 200523). Cloning was confirmed by Sanger sequencing (Eurofins Genomics).

HEK293 cells were seeded at 100,000 cells per well in 24-well plates and transfected with 20 nM control mimic or miR-147b mimic (Horizon Discovery) along with pmirGLO plasmid DNA at 0.5 μg per well (unmodified/NDUFA4 WT/NDUFA4mut) using TransIT-X2 reagent (Geneflow, E7-0106). Luciferase assays were performed using the Dual Luciferase Assay Kit (Promega, E1910) according to the manufacturer’s instructions using cell lysates harvested 1 day after transfection and a plate reader integration time of 0.1 s. Measurements were performed in technical triplicate, and the experiment was performed three separate times.

### Protein modeling

The model of C15ORF48 was built using the structure of NDUFA4 as reported in the human complex IV (*28*), according to the default energy-based homology model protocol implemented in Prime (Prime, Schrödinger, LLC, New York, NY, 2020). Unsolved atoms in side chains of residues 3 to 11, 42 to 44, and 46 to 83 of NDUFA4 were added. The completed side chains were assigned an energy minimum conformation. Sequences of NDUFA4 and C15ORF48 were retrieved from UniProt (www.uniprot.org). In light of the levels of identity (30%) and similarity (44%) between the two sequences, the default Prime pairwise sequence alignment routine was adopted. During the coordinate transfer, the cardiolipin from the experimental structure was explicitly retained. The multimeric structure encompassing C15ORF48 and all subunits from complex IV except NDUFA4 was obtained superimposing the backbone coordinates of the model to those of the template, deleting the template and minimizing the generated complex. The position and thickness of the mitochondrial membrane were assigned according to the predictions generated by the OPM (orientation of proteins in membranes) server.

### One-dimensional BN-PAGE

Mitochondrial-enriched fractions were prepared from unstimulated or LPS-treated human MDMs (1 × 10^6^ to 2 × 10^6^ cells per condition) by treatment with digitonin (4 mg/ml), centrifugation at 10,000*g* (4°C), and washing. Mitochondrial membranes were solubilized with 10% digitonin in 1.5 M aminocaproic acid, 50 mM bis-tris/HCl (pH 7.0), followed by centrifugation at 18,000*g* (4°C). BN sample buffer was added at 10% volume [0.75 M aminocaproic acid, 50 mM bis-tris/HCl (pH 7.0), 0.5 mM EDTA, 5% Coomassive Brilliant Blue G-250] and a portion of each sample run on NativePAGE 3 to 12% bis-tris gel (Thermo Fisher Scientific, BN1001) according to the manufacturer’s instructions. For Western blotting, protein was transferred to PVDF membranes by wet transfer, and protein detection was performed as described above. For in-gel activity assay, the gel was incubated overnight with complex IV activity solution as described (*77*).

### In situ hybridization and immunohistochemistry

RNAscope in situ hybridization was carried out on formalin-fixed paraffin-embedded human joint replacement tissues sections of OA and RA synovium provided by the Royal Orthopaedic Hospital (ethical approval no. 07/H1204/191). Sections were deparaffinized in xylene, followed by 100% ethanol. Detection of human *C15orf48* transcript was performed using an RNAscope 2.5 HD manual assay red kit reagents and methods according to the manufacturer (ACD Bio-Techne). Following the last wash for the RNAscope protocol, slides were washed in distilled water for 5 min and then blocked with Bloxall (Vector Laboratories) for 10 min, followed by incubation with 10% normal horse serum in tris buffer for 10 min. Immunohistochemistry was performed by incubating sections overnight at 4°C with biotin-conjugated mouse anti-human CD68 (Novus, NBP2-34661B), followed by streptavidin-HRP (Thermo Fisher Scientific) and ImmPACT DAB Peroxidase (HRP) Substrate (Vector Laboratories). Hematoxylin QS (Vector Laboratories) was used for counterstain. Images were obtained using the Zeiss Axio Scan and analyzed in Zen Blue (both Zeiss).

### Metabolic activity assays

Metabolic assays were carried out using an Agilent Seahorse XFe96 Extracellular Flux analyzer. For intact cell assays, macrophages were seeded into Seahorse XFe96 assay plates at 50,000 cells per well (human) or 75,000 (mouse), left for 1 hour at room temperature to settle, and incubated overnight to adhere. Cells were left unstimulated or stimulated with LPS (10 ng/ml) for 24 hours. For normalization of measurements, a viable cell count ratio was determined. Calcein-AM viability dye (eBioscience, 65-0853-78) at 1 μM in phosphate-buffered saline (PBS) was added to cells following assay completion, incubated at 37°C for 30 min, and fluorescence-measured by plate reader (excitation, 490 nm; emission, 515 nm).

#### Mito/Glyco stress test

A combined version of the standard Mito and Glyco stress tests was carried out as described (*78*). Assay medium is as follows: seahorse XF base medium (Agilent, 102353-100) + 2 mM glutamine. Stimulation medium was exchanged for assay medium 1 hour before running the assay. Assay injections are as follows (final assay concentrations): (i) glucose (10 mM), (ii) oligomycin (1 μM), (iii) carbonyl cyanide *p*-trifluoromethoxyphenylhydrazone (human, 5 μM; mouse, 0.5 μM) + sodium pyruvate (1 mM), and (iv) rotenone (100 nM) + antimycin A (1 μM) + 2-deoxyglucose (20 mM).

#### ATP production rate assay

Assay medium is as follows: seahorse XF Dulbecco’s modified Eagle’s medium with Hepes, without phenol red (pH 7.4) (Agilent, 103575-100) + glutamine (2 mM) + glucose (10 mM) + sodium pyruvate (1 mM). Stimulation medium was exchanged for assay medium 1 hour before running the assay. Assay injections are as follows (final assay concentrations): (i) oligomycin (1 μM), (ii) rotenone (100 nM) + antimycin A (1 μM), and (iii) 2-deoxyglucose (20 mM). ATP production rates were calculated using Agilent ATP rate assay report generator.

#### Complex IV assay

This was performed according to Salabei *et al.* (*79*). Human MDMs were seeded into seahorse XFe96 assay plates at 30,000 cells per well, left for 1 hour at room temperature to settle and incubated overnight to adhere. Cells were left unstimulated or stimulated with LPS (10 ng/ml) for 24 hours. Assay medium is as follows: buffer prepared as in (*79*) + adenosine 5′-diphosphate (1 mM) + fatty acid–free bovine serum albumin (4 mg/ml) + seahorse XF plasma membrane permeabilizer (1 nM; Agilent, 102504-100). Stimulation medium was exchanged for assay medium immediately before running the assay due to permeabilization. Assay injections are as follows (final assay concentrations): (i) *N*,*N*,*N*′,*N*′-tetramethyl-*p*-phenylenediamine (500 mM), l-ascorbic acid (200 mM); (ii) oligomycin (1 μM); (iii) rotenone (1 μM), antimycin A (1 μM); and (iv) sodium azide (20 mM). For normalization of permeabilized cells, DNA content was quantified using PicoGreen fluorescent dsDNA dye (Thermo Fisher Scientific, 41116133) after assay completion (excitation, 485 nm; emission, 528 nm).

### Mitochondrial ROS measurement

Mitochondrial ROS was detected using MitoSOX dye (5 μM; Thermo Fisher Scientific, M36008). Staining was performed in culture plates immediately following the stimulation period. Cells were washed with PBS and stained for 20 min at 37°C in Hanks’ balanced salt solution + Ca^2+^ + Mg^2+^. For positive control, antimycin A was spiked into the well 1 hour before staining. Cells were run on a BD LSRFortessa X-20, and results were analyzed using FlowJo v10. Dead cells were excluded on the basis of positive staining for eBioscience Fixable Viability Dye eFluor 780 (Thermo Fisher Scientific, 65-0865-14).

### Nitric oxide measurement

Nitrite concentration in conditioned medium from cultured macrophages was determined using the Griess reagent kit (Thermo Fisher Scientific, G7921) according to the manufacturer’s instructions. Briefly, cultured cell supernatant was collected after treatment and centrifuged to remove debris. The supernatant (100 μl) was added to 96-well plate containing 20 μl of deionized water per well and followed by the addition of an equal volume of *N*-(1-naphthyl)ethylenediamine and sulfanilic acid mixture to a total volume of 200 μl. The reaction was incubated for 30 min at room temperature. Absorbance at 548 nm was measured by microplate reader, and nitrite concentrations were estimated using a standard nitrite curve and correcting for background noise.

### NanoString gene expression analysis

Gene expression analysis from NDUFA4 patient and control macrophages was performed using the NanoString nCounter Human Inflammation Panel (NanoString, XT-CSO-HIN2) according to the manufacturer’s instructions, using 150 ng of RNA per sample. Analysis and visualization were performed using NanoString nSolver software and Qlucore Omics Explorer 3.6.

### Multiplex cytokine analysis

Conditioned medium samples from cultured macrophages were subjected to multiplex Luminex assay (Biotechne R&D Human magnetic luminex assay LXSAHM and Bio-Rad Bio-Plex Pro Human Chemokine Panel, 40-Plex 171AK99MR2) according to the manufacturer’s instructions. Differential abundance analysis and data visualization were performed using Qlucore Omics Explorer 3.6.

### Statistics

Statistical analyses were carried out using GraphPad Prism v6 unless otherwise stated. Statistical tests and corrections used are indicated in the figure legends. *P < 0.05, **P < 0.01, ***P < 0.001, and ****P < 0.0001. *N* numbers specified in the figure legends indicate biological replicates.

## Notes

View/request a protocol for this paper from *Bio-protocol*.
