# Supplementary material for: Inflammation causes remodeling of mitochondrial cytochrome c oxidase mediated by the bifunctional gene C15orf48
Source: Sci Adv. 2021 Dec 8;7(50):eabl5182. doi: 10.1126/sciadv.abl5182 (PMC8654286; doi:10.1126/sciadv.abl5182)
Supplement: Supplementary file 1 — Figs. S1 to S7 Table S1 [file sciadv.abl5182_sm.pdf]

Science Advances
AAAS

# Supplementary Materials for

## Inflammation causes remodeling of mitochondrial cytochrome *c* oxidase mediated by the bifunctional gene *C15orf48*

Sally A. Clayton*, Kalbinder K. Daley, Lucy MacDonald, Erika Fernandez-Vizarra, Giovanni Bottegoni, John D. O'Neil, Triin Major, Daniel Griffin, Qinqin Zhuang, Adeolu B. Adewoye, Kieran Woolcock, Simon W. Jones, Carl Goodyear, Aziza Elmesmari, Andrew Filer, Daniel A. Tennant, Stefano Alivernini, Christopher D. Buckley, Robert D. S. Pitceathly, Mariola Kurowska-Stolarska, Andrew R. Clark*

*Corresponding author. Email: s.clayton@bham.ac.uk (S.A.C.); a.r.clark@bham.ac.uk (A.R.C.)

Published 8 December 2021, *Sci. Adv.* **7**, eabl5182 (2021)
DOI: 10.1126/sciadv.abl5182

**This PDF file includes:**

Figs. S1 to S7
Table S1

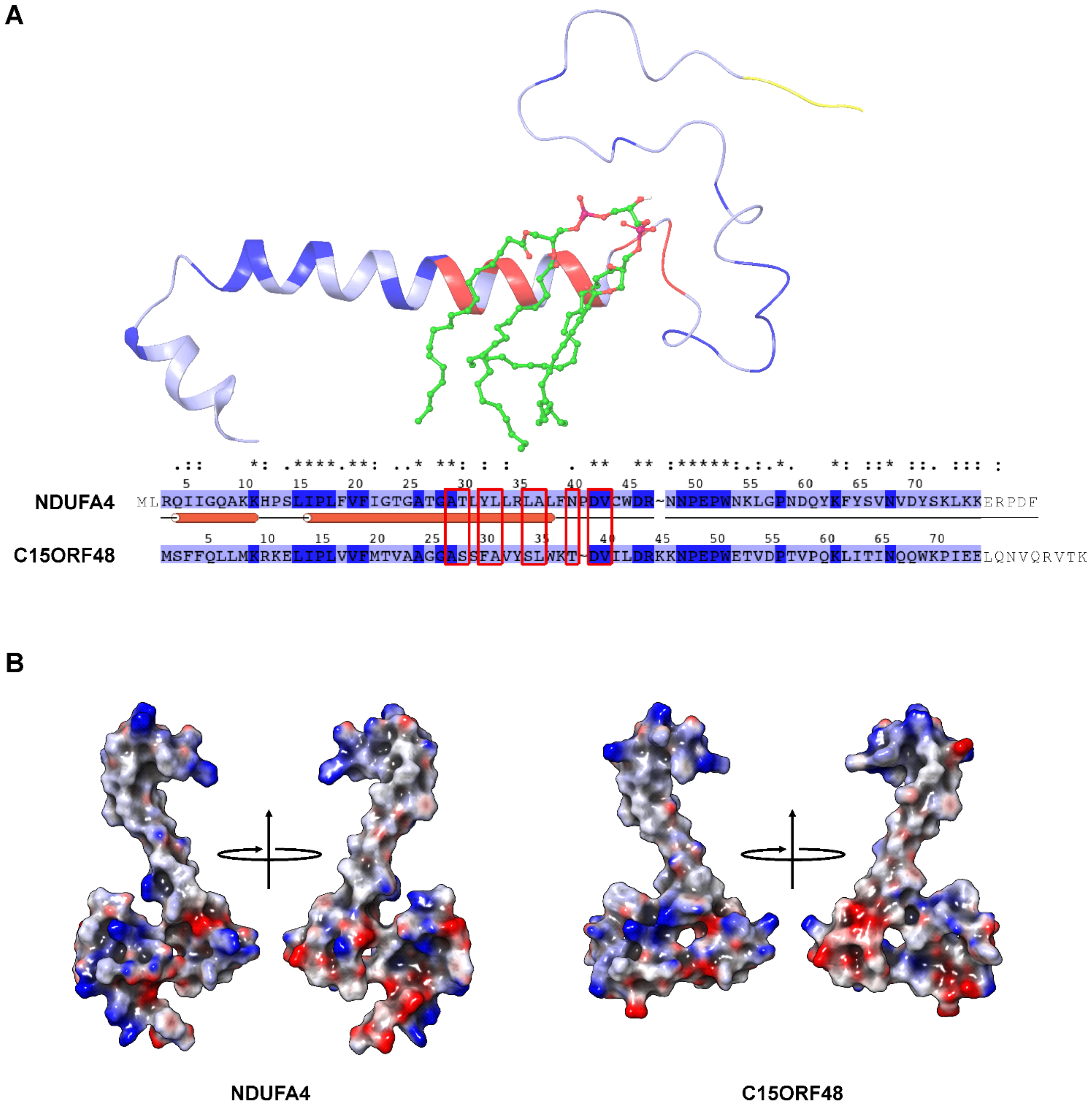


**Figure S1. C15ORF48 and NDUFA4 are structurally related despite limited sequence identity.** (**A**) the crystal structure of NDUFA4 is approximately aligned with the amino acid sequences of NDUFA4 and C15ORF48. Based on data from (*28*), the position of cardiolipin is indicated (green), showing an intimate association with the NDUFA4 alpha helix that spans the inner mitochondrial membrane. (**B**) The structure of C15ORF48 was modelled on the basis of the existing crystal structure of NDUFA4 (*28*). Electrostatic potentials of the two proteins are projected onto their Connolly surfaces.

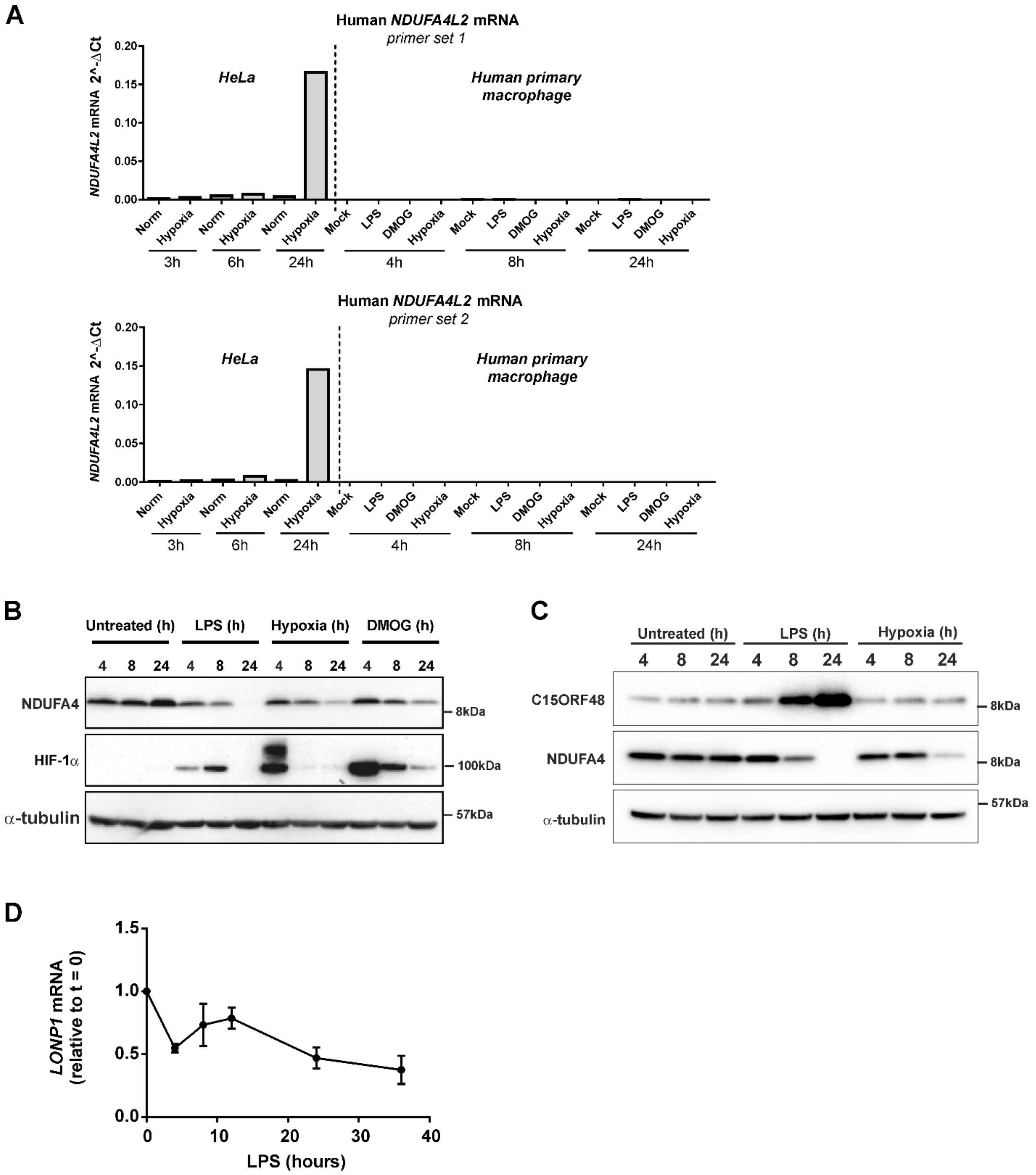


**Figure S2. Neither *Ndufa4l2* nor *C15orf48* gene is hypoxia-responsive in primary human macrophages.** (**A**) HeLa cells or MDMs were treated under hypoxia (1% $O_2$) or normoxia (20% $O_2$), stimulated with LPS or the hypoxia mimetic DMOG (dimethyloxallyl glycine) for the indicated times. *NDUFA4L2* mRNA was detected using two different primer pairs. An identical result was achieved using MDMs from a second donor. (**B**) MDMs were left untreated, exposed to hypoxia (1% $O_2$), treated with LPS or DMOG for 4, 8 or 24 h. NDUFA4, HIF-1α and α-tubulin proteins were detected by western blotting. Representative of two similar experiments. (**C**) MDMs were left untreated, exposed to hypoxia (1% $O_2$) or treated with LPS for 4, 8 or 24 h. C15ORF48, NDUFA4 and α-tubulin proteins were detected by western blotting. Representative of two similar experiments. (**D**) *LONP1* mRNA was quantified by qPCR in MDMs treated with LPS for 0 - 36h. Mean mRNA abundance ± SEM from three independent MDM cultures, normalized against the zero time point.

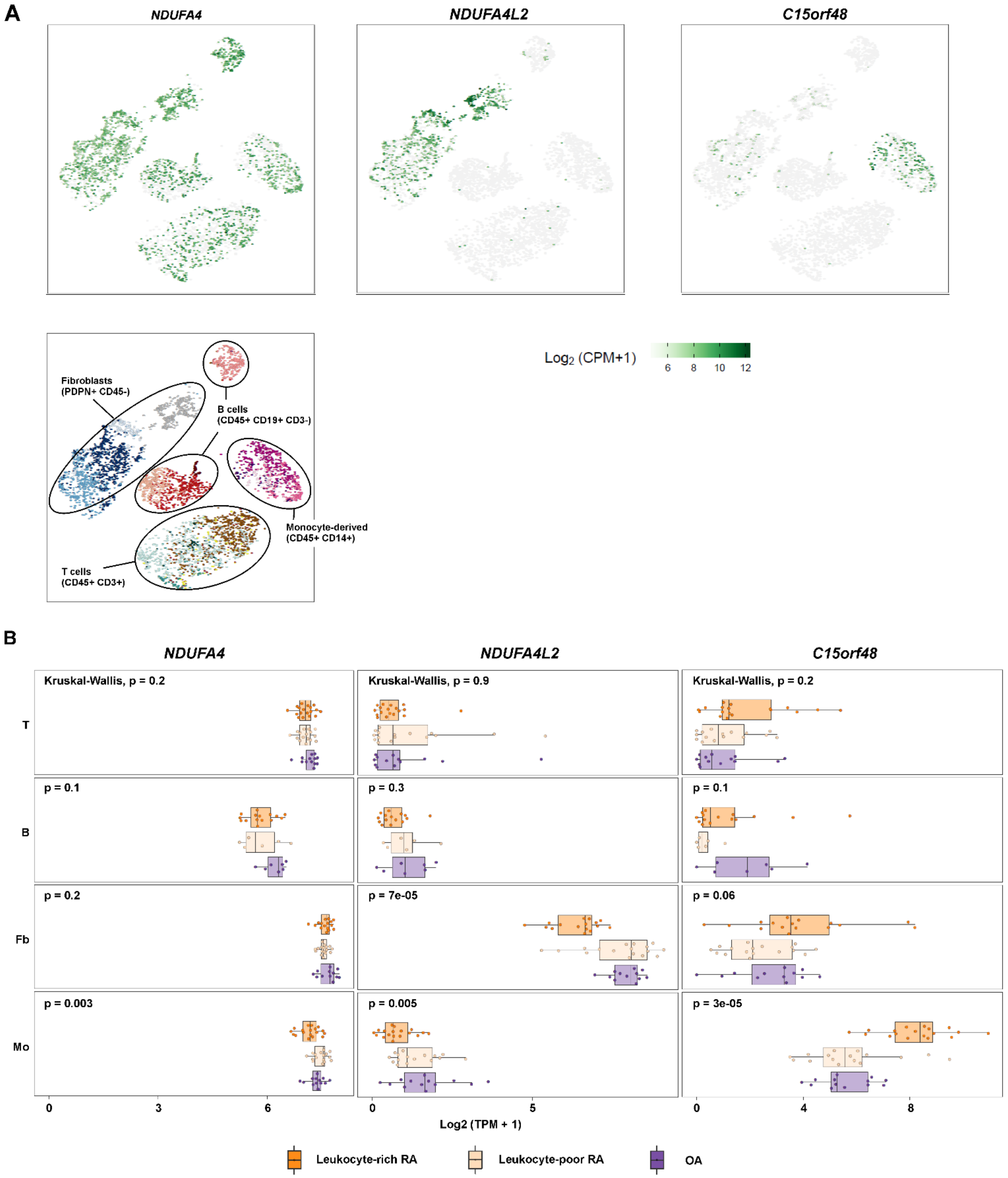


**Figure S3. Expression of *NDUFA4* family members in synovial tissue cells.** (**A**) Cells were disaggregated from synovial tissue biopsies of RA patients and subjected to single cell RNAseq (*45*). Abundances of *NDUFA4*, *NDUFA4L2* and *C15orf48* mRNAs are indicated in UMAP plots. The lower panel is the key to synovial cell populations. (**B**) Cells were disaggregated from synovial tissue biopsies of OA patients (n = 15) or RA patients classified by immunohistology as leukocyte-rich (n = 19) or leukocyte-poor (n = 17) and sorted into T cell (T), B cell (B), fibroblast (Fb) or monocyte-derived (Mo) populations using the antibodies indicated in A. Levels of *NDUFA4*, *NDUFA4L2* or *C15orf48* mRNAs were determined by bulk RNA sequencing (*45*).

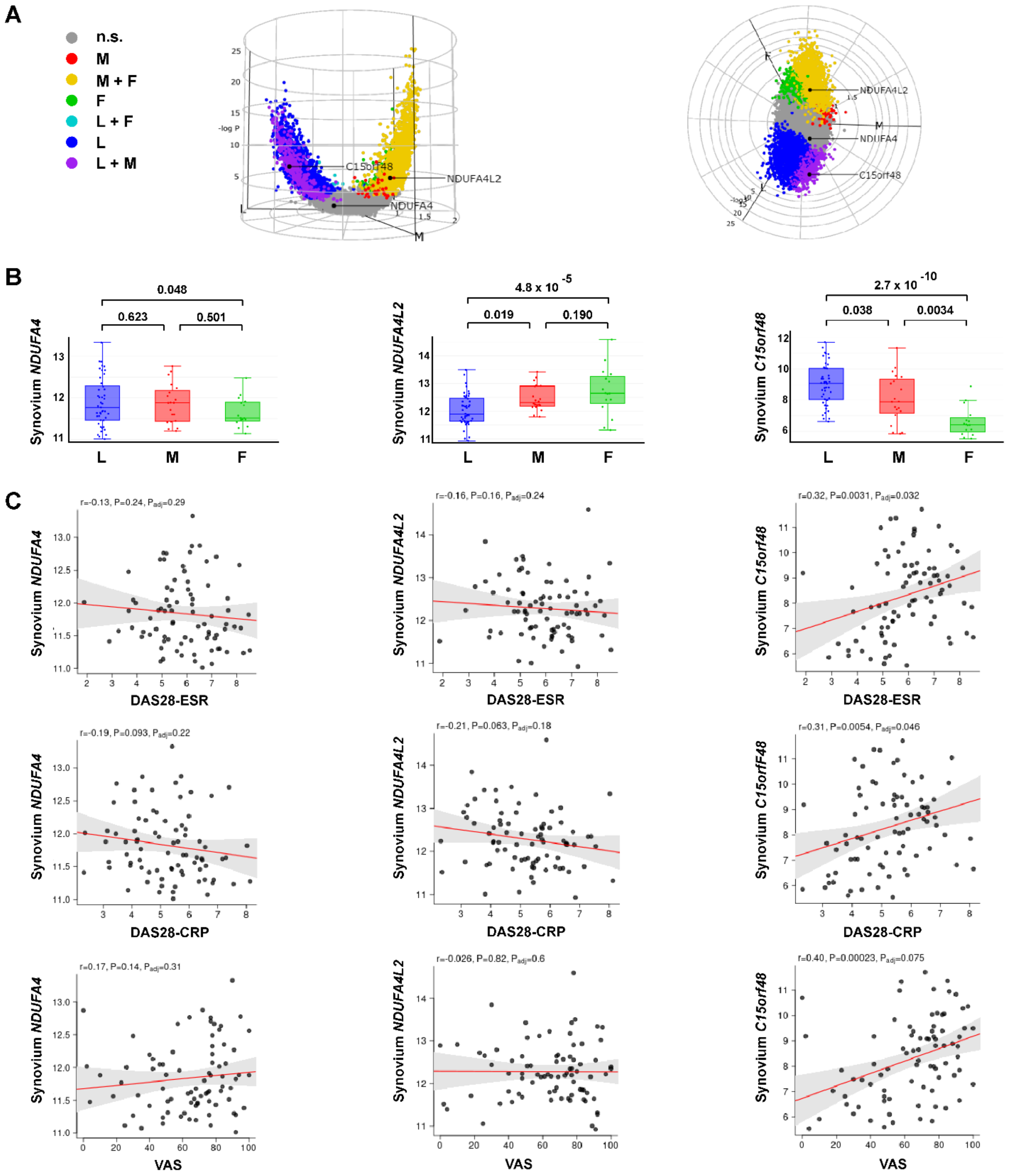


**Figure S4. Association of *C15orf48* gene expression with histologically defined RA disease pathotypes and correlation with disease activity**. (**A**) A three dimensional volcano plot was used to identify genes differentially expressed in synovial tissue of diffuse myeloid pathotype (M), pauci-immune or fibroid pathotype (F) or lymphomyeloid pathotype (L). (**B**) Abundance of *NDUFA4*, *NDUFA4L2* and *C15orf48* transcripts in synovial biopsies classified as in A. (**C**) Correlation of disease activity scores with levels of *NDUFA4*, *NDUFA4L2* and *C15orf48* transcripts in synovial biopsies. DAS28, 28 joint disease activity score; ESR, erythrocyte sedimentation rate; CRP, C reactive protein; VAS, visual analog scale. All data are from (*46*), accessed via https://peac.hpc.qmul.ac.uk/

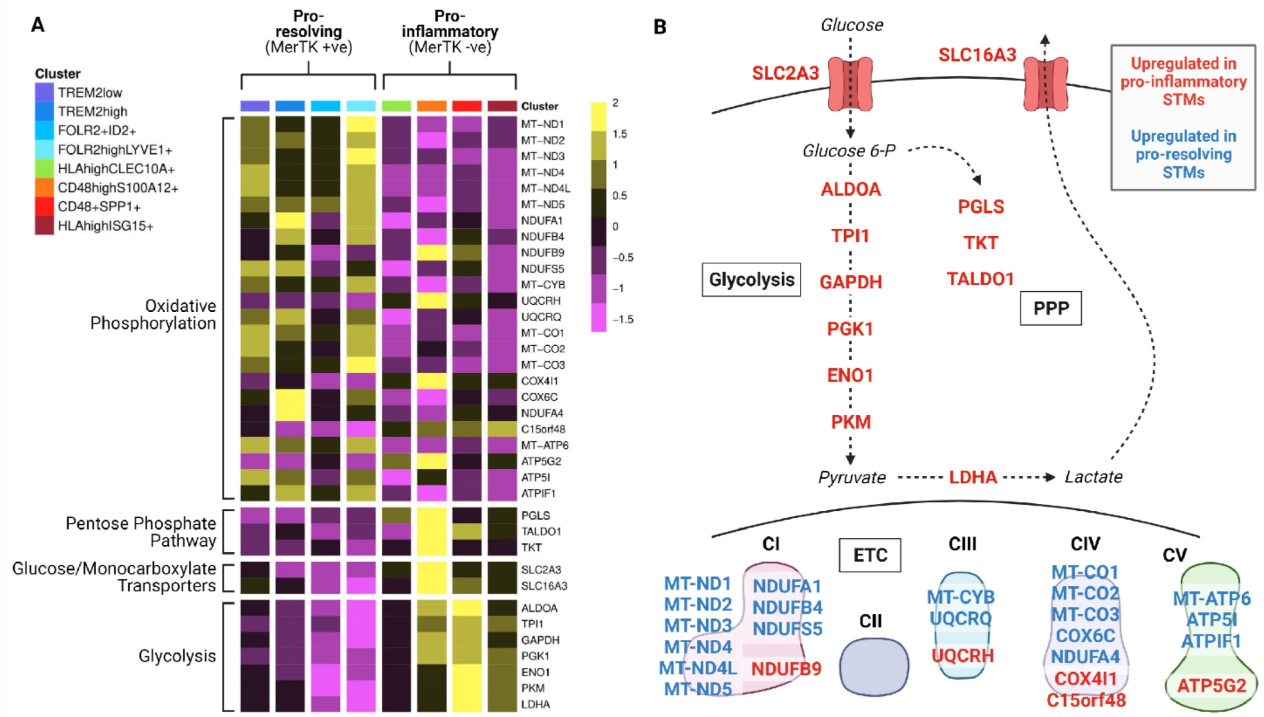


**Figure S5. Coherent patterns of expression of metabolic genes in pro- and anti-inflammatory synovial tissue macrophage populations. Reproduced from (*8*) with permission from Frontiers in Immunology.** (**A**) Heatmap of synovial tissue macrophage single cell RNA sequencing data displaying differential expression of metabolism-related genes across synovial tissue macrophage sub-populations (clusters). Detailed information on the characteristics of the populations and their contribution to different disease outcome groups can be found in (*44*). (**B**) Pathway schematic of the same data. Genes up-regulated in at least one cluster of pro-inflammatory macrophages are displayed in red, and genes up-regulated in at least one cluster of pro-resolving macrophages are displayed in blue. ETC, electron transport chain; PPP, pentose phosphate pathway.

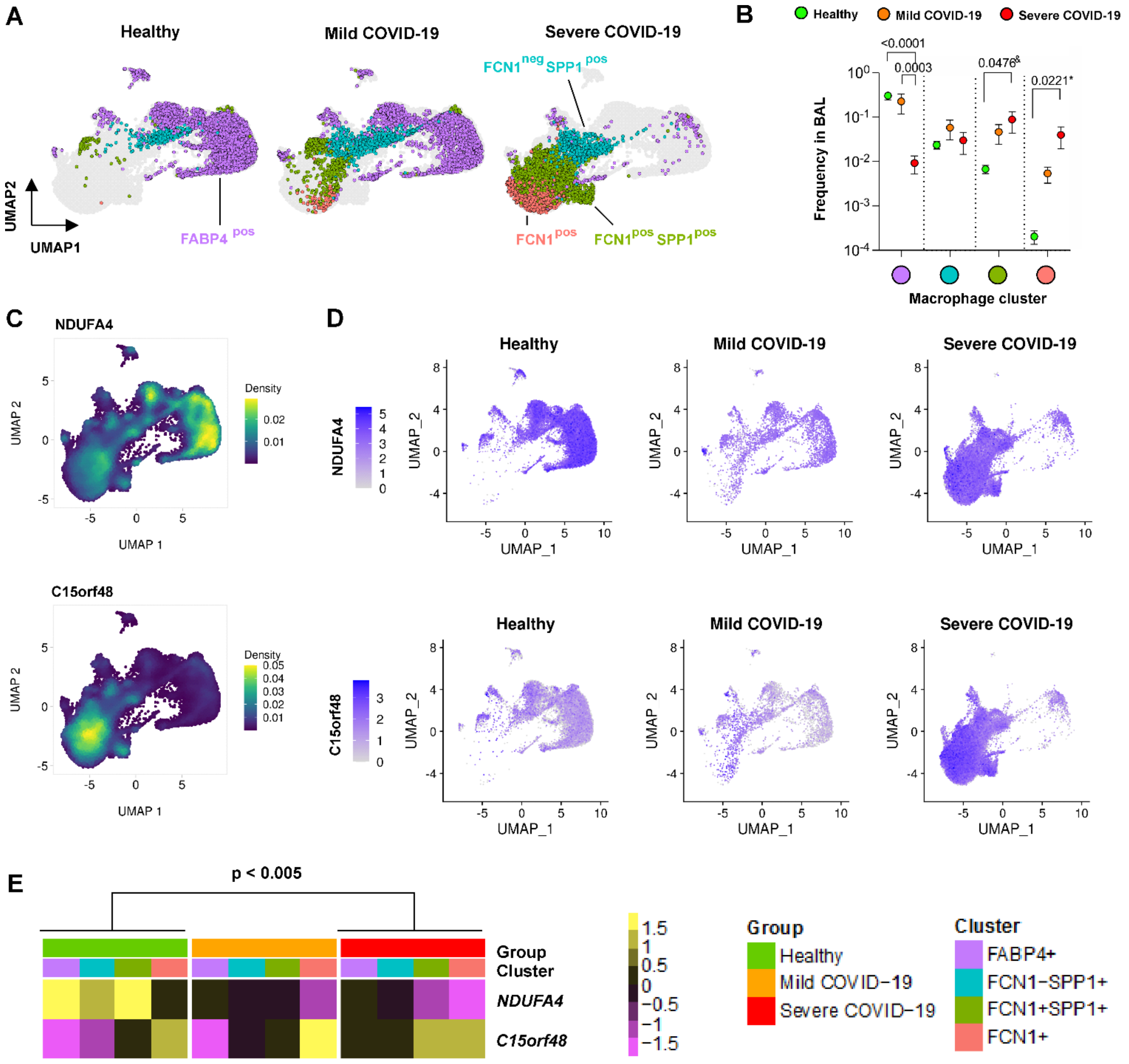


**Figure S6. Bronchoalveolar lavage fluid of severe COVID-19 patients is enriched in populations of macrophages that have pathogenic, pro-inflammatory properties and strongly express *C15orf48*.** (**A**) UMAP plots of alveolar macrophage populations in healthy controls and patients with mild or severe COVID-19 (see *51*, *52*). (**B**) Quantification of macrophage populations in the same cohort: healthy controls n=3; mild COVID-19 n=3; severe COVID-19 n=6. (**C**) Density UMAP visualization of *NDUFA4* and *C15orf48* expression. Color intensity highlights areas with high density of positive cells. (**D**) Visualization of differences in expression of *NDUFA4* and *C15orf48* across split UMAP plots. Intensity of purple indicates expression level. (**E**) Heat map illustrating scaled pseudo-bulk expression of *NDUFA4* and *C15orf48* according to patient group and macrophage cluster.

Bronchoalveolar lavage fluid of healthy controls is enriched in FABP4+ resident macrophages that strongly express *NDUFA4*. In severe COVID-19 these resident macrophages are outnumbered by FCN1+ and FCN1+SPP1+ populations that strongly express *C15orf48*.

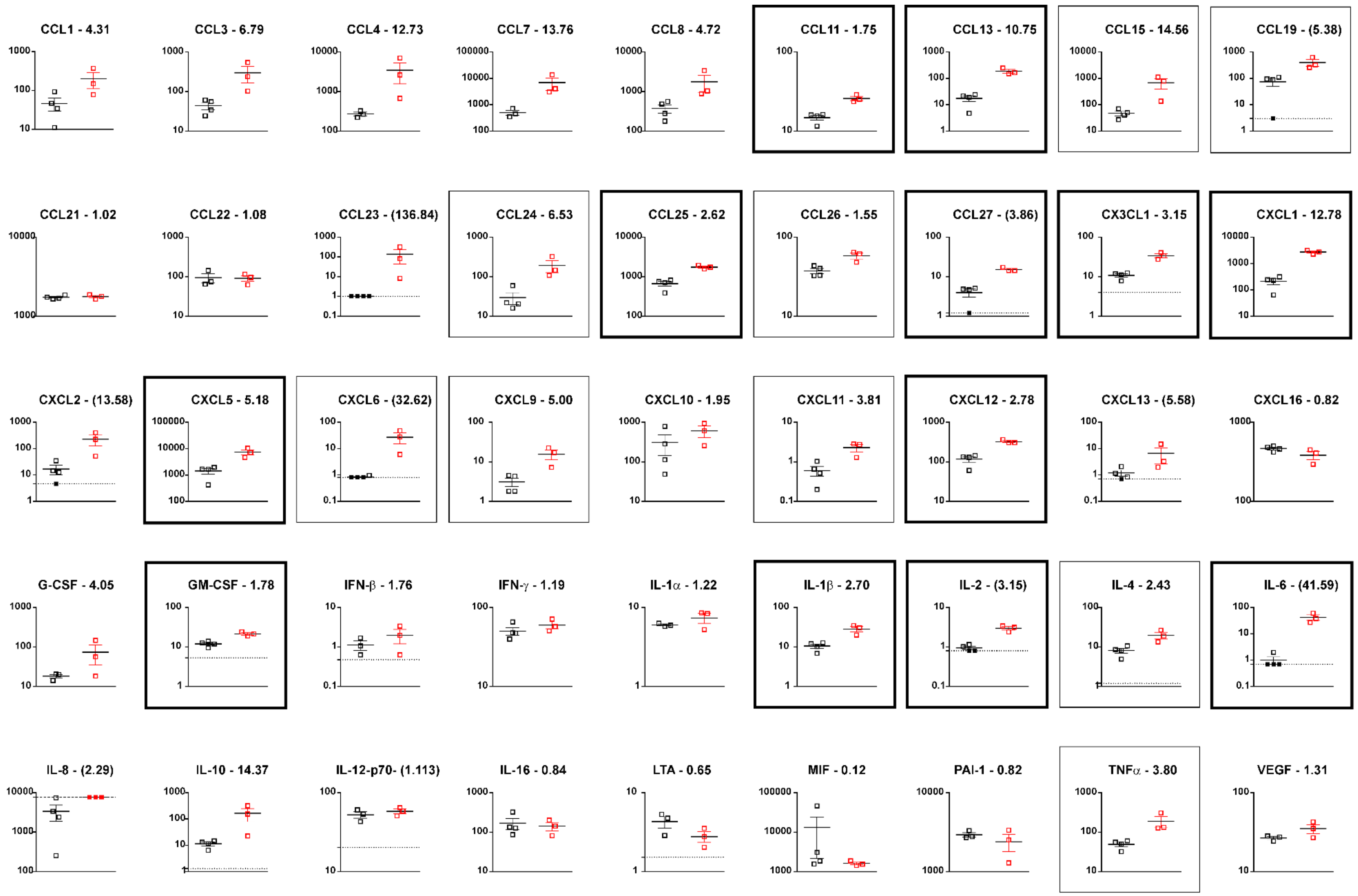


**Figure S7. Dysregulated basal cytokine and chemokine expression by NDUFA4-null macrophages.** Macrophages were derived from peripheral blood monocytes of NDUFA4-null cases (red symbols) or healthy controls (black symbols). Cytokines and chemokines in cell supernatants under resting conditions were measured by multiplexed bead assay. Light boxing, $p < 0.05$. Heavy boxing, $p < 0.01$ (two-tailed t test). Dotted lines indicate upper or lower limits of detection, where these fall within the range of y axes. Measurements above or below the limits of detection were arbitrarily assigned a value equal to the limit, and are indicated as filled boxes. Fold differences between cases and controls are shown, brackets indicating estimated fold differences (where at least one measurement was outside the assay range).

# Remodeling of mitochondria in inflammation

| Metadata | | | | | | *NDUFA4* | | *C15orf48* | |
|---|---|---|---|---|---|---|---|---|---|
| Organism | cell type | Condition | Conc/dose | Time (h) | GEO accession | adj.P.Val | log2FC | adj.P.Val | log2FC |
| Human | MDM | SA | N/A | 8 | GSE13670 | 0.57925908 | -0.224 | 0.07510444 | 0.644 |
| Human | MDM | SA | N/A | 24 | GSE13670 | 0.63801 | -0.1819034 | 0.0312265 | 0.8972223 |
| Human | MDM | SA | N/A | 48 | GSE13670 | 0.8502232 | -0.1491481 | 0.0943143 | 0.9017938 |
| Human | MDM | IFNa | 10ngmL | 4 | GSE16755 | 0.9106 | 0.13230969 | 0.4272 | 0.92757405 |
| Human | AM | H1N1 | N/A | 4 | GSE30723 | 0.34230736 | 0.23544576 | 0.84936977 | -0.0529221 |
| Human | AM | H1N1 | N/A | 24 | GSE30723 | 0.981626 | 0.00679143 | 0.017819 | 0.63097589 |
| Human | AM | LPS | 4ng/kg | 6 | GSE40885 | 0.167 | -0.171 | 0.000321 | 3.42 |
| Human | MDM | LPS | 1ngmL | 4 | GSE41295 | 0.856 | -0.1091537 | 0.000318 | 0.8491682 |
| Human | MDM | PAM | 10ugmL | 4 | GSE41295 | 0.30931058 | -0.395903 | 0.0006438 | 0.8772928 |
| Human | MDM | Poly(I:C) | 10ugmL | 4 | GSE41295 | 0.97742147 | -0.055036 | 0.69853924 | 0.232898 |
| Human | MDM | IFNa | 50UmL | 7 | GSE85333 | 0.222 | -0.328 | 0.00478 | 1.81 |
| Human | MDM | LPS | 10ngmL | 7 | GSE85333 | 0.0285 | -0.7224711 | 0.0000461 | 2.4304736 |
| Human | MDM | IFNa | 50UmL | 24 | GSE85333 | 0.641831 | -0.2512531 | 0.340863 | 1.2602868 |
| Human | MDM | LPS | 10ngmL | 24 | GSE85333 | 0.0447 | -0.48401413 | 0.000153 | 2.40500131 |
| Mouse | BMDM | IFNa | 10000UmL | 2 | GSE110549 | 0.98273713 | 0.13210314 | 0.66302229 | 0.26548768 |
| Mouse | BMDM | Poly(I:C) | 0.2ugmL | 6 | GSE124287 | 0.89229 | -0.1130827 | 0.00138 | 1.9299744 |
| Mouse | BMDM | Poly(I:C) | 1ugmL | 20 | GSE124287 | 0.36693199 | -0.20477642 | 0.0000196 | 4.22898042 |
| Mouse | Lung | Mtb | 100 CFU | 360 | GSE23014 | 0.578164 | 0.254 | 0.488583 | 3.18 |
| Mouse | Lung | Mtb | 100 CFU | 504 | GSE23014 | 0.38381378 | 0.141 | 0.0045805 | 7.72 |
| Mouse | BMDM | IFNa | 62.5UmL | 2.5 | GSE35825 | 0.76853 | -0.08212599 | 0.00575 | 2.25100222 |
| Mouse | BMDM | PAM | 1ugmL | 24 | GSE36891 | 0.58312918 | -0.08465672 | 0.00003984 | 3.10860062 |
| Mouse | BMDM | Poly(I:C) | 2ugmL | 24 | GSE36891 | 0.00241 | -0.74404716 | 0.000000578 | 4.67904615 |
| Mouse | PM | LPS | 10ngmL | 24 | GSE42190 | 0.00564 | -0.71130394 | 0.000000197 | 4.85659938 |
| Mouse | PM | LPS | 10ngmL | 24 | GSE42190 | 0.287 | -0.29297127 | 0.00000297 | 4.59939315 |
| Mouse | AM | H1N1 | N/A | 48 | GSE42639 | 0.999963 | 0.15514663 | 0.1553851 | 1.0740145 |
| Mouse | AM | H1N1 PR8 | 100LD50 | 48 | GSE42639 | 0.0996 | -0.42439142 | 3.17E-09 | 4.930778 |
| Mouse | AM | H1N1 PR8 | 10LD50 | 48 | GSE42639 | 0.756 | -0.1601378 | 0.00000693 | 4.72761647 |
| Mouse | AM | H1N1 PR8 | 0.6LD50 | 72 | GSE42639 | 0.177 | -0.33357428 | 0.0000508 | 3.62381029 |
| Mouse | BMDM | IFNb | 10UmL | 1 | GSE44292 | 0.8701 | -0.1390265 | 0.6551 | 0.5915895 |
| Mouse | BMDM | LPS | 0.5ngmL | 1 | GSE44292 | 0.508 | 0.3017675 | 0.386 | -0.8681275 |
| Mouse | BMDM | LPS | 50ngmL | 1 | GSE44292 | 0.5932 | 0.195483 | 0.5313 | -0.763733 |
| Mouse | BMDM | LPS | 5ngmL | 1 | GSE44292 | 0.8048 | -0.06777 | 0.19775 | 0.723435 |
| Mouse | BMDM | LPS | 5ngmL | 1 | GSE44292 | 0.89881 | -0.08087 | 0.1669 | -1.412833 |
| Mouse | BMDM | IFNb | 10UmL | 2 | GSE44292 | 0.52723 | -0.249134 | 0.11736 | 0.9457375 |
| Mouse | BMDM | LPS | 0.5ngmL | 2 | GSE44292 | 0.825 | -0.0684005 | 0.104 | -1.4576335 |
| Mouse | BMDM | LPS | 50ngmL | 2 | GSE44292 | 0.6878 | 0.0897975 | 0.3562 | -0.469281 |
| Mouse | BMDM | LPS | 5ngmL | 2 | GSE44292 | 0.73152 | 0.181 | 0.04312 | 0.8316055 |
| Mouse | BMDM | LPS | 5ngmL | 2 | GSE44292 | 0.2525 | -0.319414 | 0.34533 | -0.593 |
| Mouse | BMDM | IFNb | 10UmL | 4 | GSE44292 | 0.783197 | 0.042335 | 0.001722 | 2.3903785 |
| Mouse | BMDM | LPS | 0.5ngmL | 4 | GSE44292 | 0.8036 | -0.0518155 | 0.3004 | -0.431348 |
| Mouse | BMDM | LPS | 50ngmL | 4 | GSE44292 | 0.5855 | 0.2497975 | 0.1294 | 1.2524525 |
| Mouse | BMDM | LPS | 5ngmL | 4 | GSE44292 | 0.976116 | -0.0071645 | 0.002763 | 2.849594 |
| Mouse | BMDM | LPS | 5ngmL | 4 | GSE44292 | 0.9474 | -0.0147 | 0.1993 | 0.767 |
| Mouse | BMDM | IFNb | 10UmL | 8 | GSE44292 | 0.63235 | -0.1071565 | 0.00437 | 2.1044645 |
| Mouse | BMDM | LPS | 0.5ngmL | 8 | GSE44292 | 0.5995 | 0.1523955 | 0.0728 | 0.937835 |

| | | | | | | | | | |
|---|---|---|---|---|---|---|---|---|---|
| Mouse | BMDM | LPS | 50ngmL | 8 | GSE44292 | 0.41078 | 0.1404075 | 0.00354 | 2.9466035 |
| Mouse | BMDM | LPS | 5ngmL | 8 | GSE44292 | 0.60619 | 0.0831365 | 0.00499 | 2.6823735 |
| Mouse | BMDM | LPS | 5ngmL | 8 | GSE44292 | 0.16557 | -0.2686235 | 0.001071 | 4.103078 |
| Mouse | BMDM | LPS | 0.5ngmL | 24 | GSE44292 | 0.31338 | 0.271268 | 0.00986 | 1.984598 |
| Mouse | BMDM | LPS | 50ngmL | 24 | GSE44292 | 0.16437 | -0.262614 | 0.01287 | 1.688203 |
| Mouse | BMDM | LPS | 5ngmL | 24 | GSE44292 | 0.9473 | 0.033231 | 0.001304 | 3.9675415 |
| Mouse | BMDM | LPS | 5ngmL | 24 | GSE44292 | 0.163111 | -0.512738 | 0.01322 | 1.955724 |
| Mouse | BMDM | LPS | 10ugmL | 24 | GSE45054 | 0.018152 | -0.708 | 0.00241259 | 3.37112538 |
| Mouse | BMDM | poly(I:C) | 6ugmL | 6 | GSE53986 | 0.0787 | -0.36186625 | 0.000121 | 5.28 |
| Mouse | BMDM | poly(I:C) | 6ugmL | 18 | GSE54064 | 0.00018 | -1.11 | 0.0000018 | 5.52325284 |
| Mouse | BMDM | poly(I:C) | 6ugmL | 18 | GSE54064 | 0.00018 | -1.11 | 0.000000244 | 6.55 |
| Mouse | BMDM | LPS | 10ugmL | 6 | GSE54064 | 0.0255 | -0.50981956 | 0.000000244 | 6.55 |
| Mouse | BMDM | IFNb | 10UmL | 18 | GSE60290 | 0.75836608 | -0.16041059 | 8.19E-08 | 5.64816042 |
| Mouse | PM | LPS | 1ugmL | 4 | GSE60290 | 0.98757883 | 0.00275155 | 0.7690806 | 0.19631762 |
| Mouse | BMDM | CpG | 100UmL | 4 | GSE81291 | 0.597932 | -0.34477115 | 0.252406 | 2.81811268 |
| Mouse | BMDM | LPS | 10ugmL | 4 | GSE81291 | 0.285943 | -0.5378848 | 0.123779 | 2.3532947 |
| Mouse | BMDM | PAM | 10ugmL | 4 | GSE81291 | 0.961 | -0.111 | 0.892 | 1.37 |
| Mouse | BMDM | Poly(I:C) | 10ugmL | 4 | GSE81291 | 0.97083 | 0.02563805 | 0.98013 | 0.05041488 |
| Mouse | BMDM | CpG | 100UmL | 8 | GSE81291 | 0.834332 | -0.11877229 | 0.466493 | 1.11911834 |
| Mouse | BMDM | LPS | 10ugmL | 8 | GSE81291 | 0.6329 | -0.30350434 | 0.2803 | 2.87713043 |
| Mouse | BMDM | PAM | 100ngmL | 8 | GSE81291 | 0.961 | -0.111 | 0.892 | 1.37 |
| Mouse | BMDM | Poly(I:C) | 10ugmL | 8 | GSE81291 | 0.680731 | -0.32300023 | 0.441093 | 2.94064313 |
| Mouse | BMDM | Poly(I:C) | 250ugmL | 0.5 | GSE89988 | 0.647256 | -0.26358008 | 0.647256 | 1.64529681 |
| Mouse | BMDM | Poly(I:C) | 250ugmL | 0.5 | GSE89988 | 0.0162 | -0.45 | 0.0206 | 2.51 |
| Mouse | BMDM | LPS | 10ugmL | 1 | GSE89988 | 0.504 | -0.1635359 | 0.763 | -0.3938573 |
| Mouse | BMDM | PAM | 10ugmL | 1 | GSE89988 | 0.924342 | -0.0428 | 0.8534075 | -0.623 |
| Mouse | BMDM | Poly(I:C) | 250ugmL | 1 | GSE89988 | 0.826 | -0.20294565 | 1 | 0.27375381 |
| Mouse | BMDM | LPS | 10ugmL | 4 | GSE89988 | 0.00261 | -0.52503086 | 0.000379 | 3.69071655 |
| Mouse | BMDM | Poly(I:C) | 250ugmL | 4 | GSE89988 | 0.0041 | -0.39007786 | 0.244 | 1.10699378 |
| Mouse | BMDM | LPS | 10ugmL | 8 | GSE89988 | 0.00421 | -0.50358482 | 0.000168 | 4.02056677 |
| Mouse | BMDM | PAM | 10ugmL | 8 | GSE89988 | 0.184 | -0.17647033 | 0.0000205 | 1.52144669 |
| Mouse | BMDM | Poly(I:C) | 250ugmL | 8 | GSE89988 | 0.822 | -0.06128066 | 0.00559 | 2.04318237 |
| Mouse | BMDM | Poly(I:C) | 250ugmL | 2 | GSE92761 | 0.628 | -0.18970923 | 0.58 | 1.32224526 |
| Mouse | BMDM | Poly(I:C) | 250ugmL | 2 | GSE92761 | 0.628 | -0.18970923 | 0.58 | 1.32224526 |

**Table S1:** Relates to Fig. 3F. Expression of *NDUFA4* and *C15orf48* mRNAs in myeloid cells, derived from Gene Expression Omnibus data. Abbreviations: AM, alveolar macrophages; BMDM, bone marrow-derived macrophages; CFU, colony-forming units; CpG, CpG-rich oligonucleotide; H1N1, influenza virus (PR8 is a highly pathogenic strain); IFNa, Interferon $\alpha$; IFNb, Interferon $\beta$; LPS, lipopolysaccharide; Mtb, Mycobacterium tuberculosis; PAM, lipopeptide (S)-(2,3-bis(palmitoyloxy)-(2RS)-propyl)-N-palmitoyl-(R)-Cys-(S)-Ser(S)-Lys(4)-OH; PM, peritoneal macrophage; Poly(I:C), polyinosinic:polycytidylic acid; SA, Staphylococcus aureus.
